# Synthetic biology for the directed evolution of protein biocatalysts: navigating sequence space intelligently

**DOI:** 10.1039/c4cs00351a

**Published:** 2014-12-15

**Authors:** Andrew Currin, Neil Swainston, Philip J. Day, Douglas B. Kell

**Affiliations:** a Manchester Institute of Biotechnology , The University of Manchester , 131, Princess St , Manchester M1 7DN , UK . Email: dbk@manchester.ac.uk ; http://dbkgroup.org/; @dbkell ; Tel: +44 (0)161 306 4492; b School of Chemistry , The University of Manchester , Manchester M13 9PL , UK; c Centre for Synthetic Biology of Fine and Speciality Chemicals (SYNBIOCHEM) , The University of Manchester , 131, Princess St , Manchester M1 7DN , UK; d School of Computer Science , The University of Manchester , Manchester M13 9PL , UK; e Faculty of Medical and Human Sciences , The University of Manchester , Manchester M13 9PT , UK

## Abstract

The amino acid sequence of a protein affects both its structure and its function. Thus, the ability to modify the sequence, and hence the structure and activity, of individual proteins in a systematic way, opens up many opportunities, both scientifically and (as we focus on here) for exploitation in biocatalysis. Modern methods of synthetic biology, whereby increasingly large sequences of DNA can be synthesised *de novo*, allow an unprecedented ability to engineer proteins with novel functions. However, the number of possible proteins is far too large to test individually, so we need means for navigating the ‘search space’ of possible protein sequences efficiently and reliably in order to find desirable activities and other properties. Enzymologists distinguish binding (*K*_d_) and catalytic (*k*_cat_) steps. In a similar way, judicious strategies have blended design (for binding, specificity and active site modelling) with the more empirical methods of classical directed evolution (DE) for improving *k*_cat_ (where natural evolution rarely seeks the highest values), especially with regard to residues distant from the active site and where the functional linkages underpinning enzyme dynamics are both unknown and hard to predict. Epistasis (where the ‘best’ amino acid at one site depends on that or those at others) is a notable feature of directed evolution. The aim of this review is to highlight some of the approaches that are being developed to allow us to use directed evolution to improve enzyme properties, often dramatically. We note that directed evolution differs in a number of ways from natural evolution, including in particular the available mechanisms and the likely selection pressures. Thus, we stress the opportunities afforded by techniques that enable one to map sequence to (structure and) activity *in silico*, as an effective means of modelling and exploring protein landscapes. Because known landscapes may be assessed and reasoned about as a whole, simultaneously, this offers opportunities for protein improvement not readily available to natural evolution on rapid timescales. Intelligent landscape navigation, informed by sequence-activity relationships and coupled to the emerging methods of synthetic biology, offers scope for the development of novel biocatalysts that are both highly active and robust.

## Abstract 2

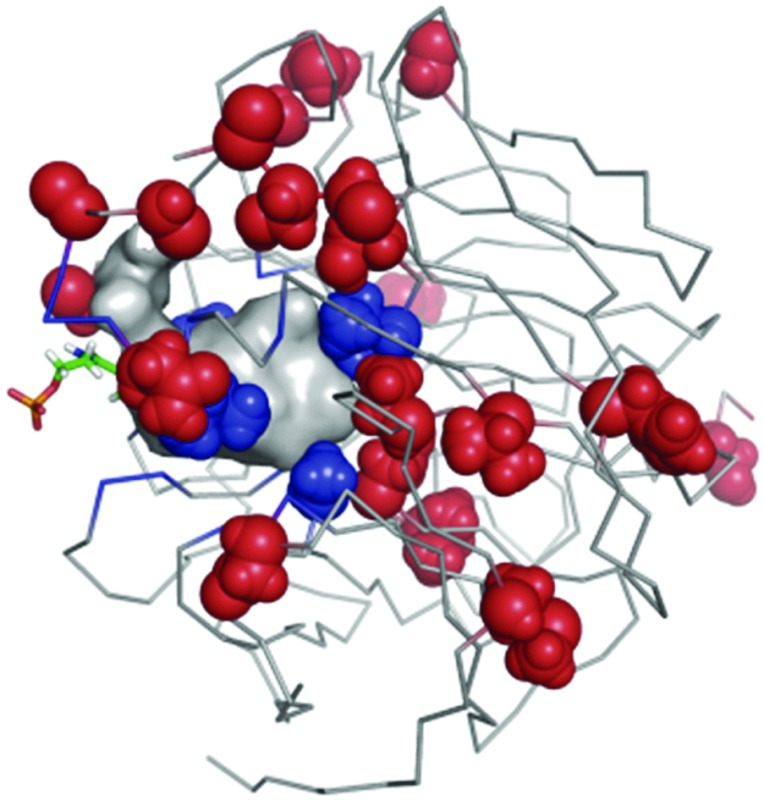
Improving enzymes by directed evolution requires the navigation of very large search spaces; we survey how to do this intelligently.

## Introduction

**Fig. 1:**
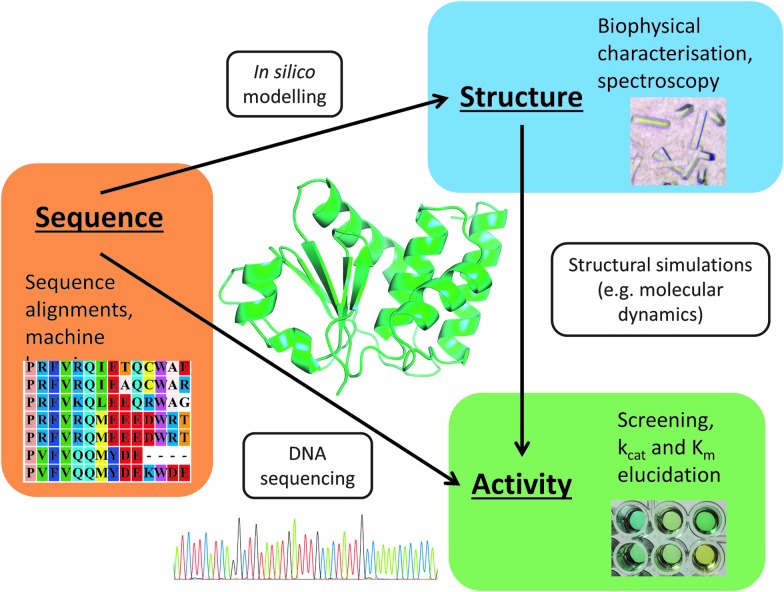
Relationship between amino acid sequence, 3D structure (and dynamics) and biocatalytic activity. Implicitly, there is a host in which these manipulations take place (or they may be done entirely *in vitro*). This is not a major focus of the review. Typically, a directed evolution study concentrates on the relationships between protein sequence, structure and activity, and the usual means for assessing these are outlined (within the boxes). Many methods are available to connect and rationalise these relationships and some examples are shown (grey boxes). Thorough directed evolution studies require understanding of each of these parameters so that the changes in protein function can be rationalised, thereby to allow effective search of the sequence space. The key is to use emerging knowledge from multiple sources to navigate the search spaces that these represent. Although the same principles apply to multi-subunit proteins and protein complexes, most of what is written focuses on single-domain proteins that, like ribonuclease,^1342,1343^ can fold spontaneously into their tertiary structures without the involvement of other proteins, chaperones, *etc.*

Much of science and technology consists of the search for desirable solutions, whether theoretical or realised, from an enormously larger set of possible candidates. The design, selection and/or improvement of biomacromolecules such as proteins represents a particularly clear example.^1^ This is because natural molecular evolution is caused by changes in protein primary sequence that (leaving aside other factors such as chaperones and post-translational modifications) can then fold to form higher-order structures with altered function or activity; the protein then undergoes selection (positive or negative) based on its new function (Fig. 1). Bioinformatic analyses can trace the path of protein evolution at the sequence level^2–4^ and match this to the corresponding change in function.

**Fig. 2:**
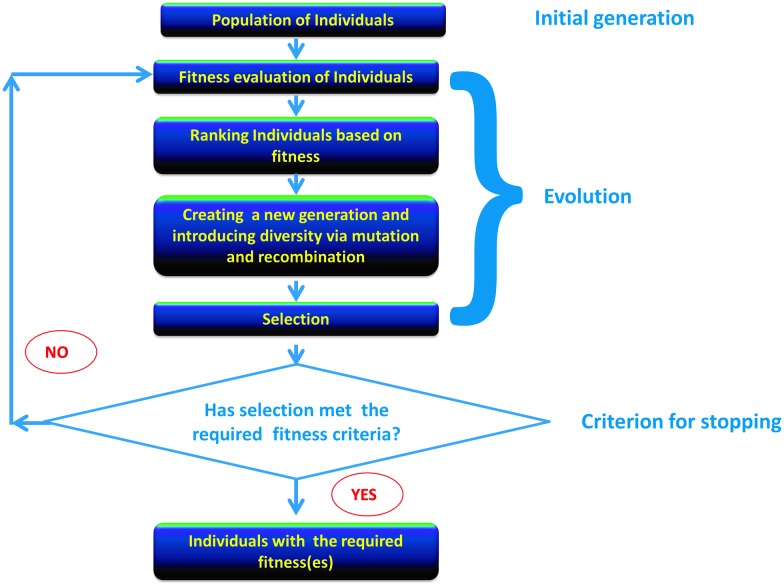
The essential components of an evolutionary system. At the outset, a starting individual or population is selected, and one or more fitness criteria that reflect the objective of the process are determined. Next, the ability to rank these fitnesses and to select for diversity is created (by breeding individuals with variant sequences, introduced typically by mutation and/or recombination) in a way that tends to favour fitter individuals, this is repeated iteratively until a desired criterion is met.

Proteins are nature's primary catalysts, and as the unsustainability of the present-day hydrocarbon-based petrochemicals industry becomes ever more apparent, there is a move towards carbohydrate feedstocks and a parallel and burgeoning interest in the use of proteins to catalyse reactions of non-natural as well as of natural chemicals. Thus, as well as observing the products of natural evolution we can now also initiate changes, whether *in vivo* or *in vitro*, for any target sequence. When the experimenter has some level of control over what sequence is made, variations can be introduced, screened and selected over several iterative cycles (‘generations’), in the hope that improved variants can be created for a particular target molecule, in a process usually referred to as directed evolution (Fig. 2) or DE. Classically this is achieved in a more or less random manner or by making a small number of specific changes to an existing sequence (see below); however, with the emergence of ‘synthetic biology’ a greater diversity of sequences can be created by assembling the desired sequence *de novo* (without a starting template to amplify from). Hence, almost any bespoke DNA sequence can be created, thus permitting the engineering of biological molecules and systems with novel functions. This is possible largely due to the reducing cost of DNA oligonucleotide synthesis and improvements in the methods that assemble these into larger fragments and even genomes.^5,6^ Therefore, the question arises as to what sequences one should make for a particular purpose, and on what basis one might decide these sequences.

**Fig. 3:**
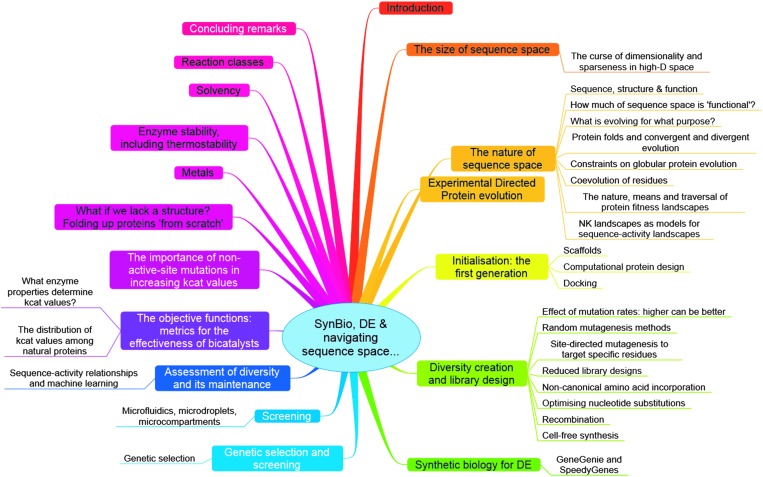
A ‘mind map’^1344^ of the contents of this paper; to read this start at “twelve o'clock” and read clockwise.

**Fig. 4:**
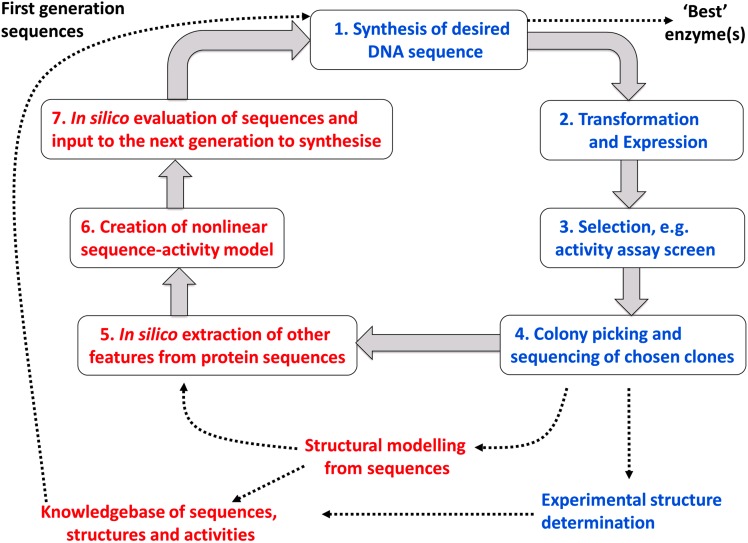
An example of the basic elements of a mixed computational and experimental programme in directed evolution. Implicit are the choice of objective function (*e.g.* a particular catalytic activity with a certain turnover number) and the starting sequences that might be used with an initial or ‘wild type’ activity from which one can evolve improved variants. The core experimental (blue) and computational (red) aspects are shown as seven steps of an iterative cycle involving the creation and analysis of appropriate protein sequences and their attendant activities. Additional facets that can contribute to the programme are also shown (connected using dotted lines).

In this intentionally wide-ranging review, we introduce the basis of protein evolution (sequence spaces, constraints and conservation), discuss the methodologies and strategies that can be utilised for the directed evolution of individual biocatalysts, and reflect on their applications in the recent literature. To restrict our scope somewhat, we largely discount questions of the directed evolution of pathways (*i.e.* series of reactions) or gene clusters (*e.g.* ref. 7 and 8) and of the choice^9^ or optimization of the host organism or expression conditions in which such directed evolution might be performed or its protein products expressed, nor the process aspects of any fermentation or biotransformation. We also focus on catalytic rate constants, albeit we recognize the importance of enzyme stability as well. Most of the strategies we describe can equally well be applied to proteins whose function is not directly catalytic, such as vaccines, binding agents, and the like. Consequently we intend this review to be a broadly useful resource or portal for the entire community that has an interest in the directed evolution of protein function. A broad summary is given as a mind map in Fig. 3, while the various general elements of a modern directed evolution program, on which we base our development of the main ideas, appears as Fig. 4.

## The size of sequence space

An important concept when considering a protein's amino acid sequence is that of (its) sequence space, *i.e.* the number of variations of that sequence that can possibly exist. Straightforwardly, for a protein that contains just the 20 main natural amino acids, a sequence length of *N* residues has a total number of possible sequences of 20^*N*^. For *N* = 100 (a rather small protein) the number 20^100^ (∼1.3 × 10 ^130^) is already far greater than the number of atoms in the known universe. Even a library with the mass of the Earth itself – 5.98 × 10^27^ g – would comprise at most 3.3 × 10^47^ different sequences, or a miniscule fraction of such diversity.^10^ Extra complexity, even for single-subunit proteins, also comes with incorporation of additional structural features beyond the primary sequence, like disulphide linkages, metal ions,^11^ cofactors and post-translational modifications, and the use of non-standard amino acids (outwith the main 20). Beyond this, there may be ‘moonlighting’ activities^12^ by which function is modified *via* interaction with other binding partners.

Considering sequence variation, using only the 20 ‘common’ amino acids, the number of sequence variants for *M* substitutions in a given protein of *N* amino acids is 
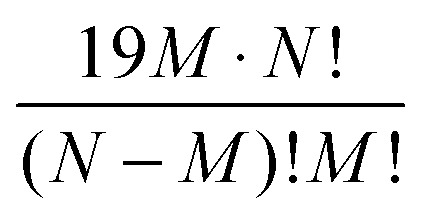
.^13^ For a protein of 300 amino acids with random changes in just 1, 2 or 3 amino acids in the whole protein this is 5700, *ca.* 16 million and *ca.* 30 billion, while even for a comparatively small protein of *N* = 100 amino acids, the number of variants exceeds 10^15^ when *M* = 10. Insertions can be considered as simply increasing the length of *N* and the number of variants to 21 (a ‘gap’ being coded as a 21st amino acid), respectively.

Consequently, the search for variants with improved function in these large sequence spaces is best treated as a combinatorial optimization problem,^1^ in which a number of parameters must be optimised simultaneously to achieve a successful outcome. To do this, heuristic strategies (that find good but not provably optimal solutions) are appropriate; these include algorithms based on evolutionary principles.

### The ‘curse of dimensionality’ and the sparseness or ‘closeness’ of strings in sequence space

One way to consider protein sequences (or any other strings of this type) is to treat each position in the string as a dimension in a discrete and finite space. In an elementary way, an amino acid X has one of 20 positions in 1-dimensional space, an individual dimer X_*k*_Y_*l*_ has a specified position or represents a point (from 400 discrete possibilities) in 2D space, a trimer X_*k*_Y_*l*_Z_*m*_ a specified location (from 8000) in 3D space, and so on. Various difficulties arise, however (‘the curse of dimensionality’^14,15^) as the number of dimensions increases, even for quite small numbers of dimensions or string length, since the dimensionality increases exponentially with the number of residues being changed. One in particular is the potential ‘closeness’ to each other of various randomly selected sequences, and how this effectively diverges extremely rapidly as their length is increased.

Imagine (as in ref. 16) that we have examples uniformly distributed in a p-dimensional hypercube, and wish to surround a target point with a hypercubical ‘neighbourhood’ to capture a fraction *r* of all the samples. The edge length of the (hyper)cube will be *e*_*p*_(*r*) = *r* ^(1/*p*)^. In just 10 dimensions *e*_10_(0.01) = 0.63 and *e*_10_(0.1) = 0.79 while the range (of a unit hypercube) for each dimension is just 1. Thus to capture even just 1% or 10% of the observations we need to cover 63% or 80% of the range (*i.e.* values) of each individual dimension. Two consequences for any significant dimensionality are that even large numbers of samples cover the space only very sparsely indeed, and that most samples are actually close to the edge of the n-dimensional hypercube. We shall return later to the question of metrics for the effective distance between protein strings and for the effectiveness of protein catalysts; for the latter we shall assume (and discuss below) that the enzyme catalytic rate constant or turnover number (with units of s^–1^, or in less favourable cases min^–1^, h^–1^, or d^–1^) is a reasonable surrogate for most functional purposes.

Overall, it is genuinely difficult to grasp or to visualise the vastness of these search spaces,^17^ and the manner in which even very large numbers of examples populate them only extremely sparsely. One way to visualise them^18–22^ is to project them into two dimensions. Thus, if we consider just 30mers of nucleic acid sequences, and in which each position can be A, T, G or C, the number of possible variants is 4^30^, which is ∼10^18^, and even if arrayed as 5 μm spots the array would occupy 29 km^2^!^23^ The equivalent array for proteins would contain only 14mers, in that there are more than 10^18^ possible proteins containing the 20 natural amino acids when their length is just 14 amino acids.

## The nature of sequence space

### Sequence, structure and function

One of the fundamental issues in the biosciences is the elucidation of the relationship between a protein's primary sequence, its structure and its function. Difficulties arise because the relationship between a protein's sequence and structure is highly complex, as is the relationship between structure and function. Even single mutations at an individual residue can change a protein's activity completely – hence the discovery of ‘inborn errors of metabolism’.^24,25^ (The same is true in pharmaceutical drug discovery, with quite small changes in small molecule structure often leading to a dramatic change in activity – so-called ‘activity cliffs’^26–33^ – and with similar metaphors of structure–activity relationships, rather than those of sequence-activity, being equally explicit.^34–37^) Annotation of putative function from unknown sequences is largely based upon sequence homology (similarity) to proteins of known characterised function and particularly the presence of specific sequence/structure motifs (such as the Rossmann fold^38^ or the P-loop motif^39^). While there have been great advances in predicting protein structure from primary sequence (see later), the prediction of function from structure (let alone sequence) remains an important (if largely unattained) aim.^40–54^

### How much of sequence space is ‘functional’?

**Fig. 5:**
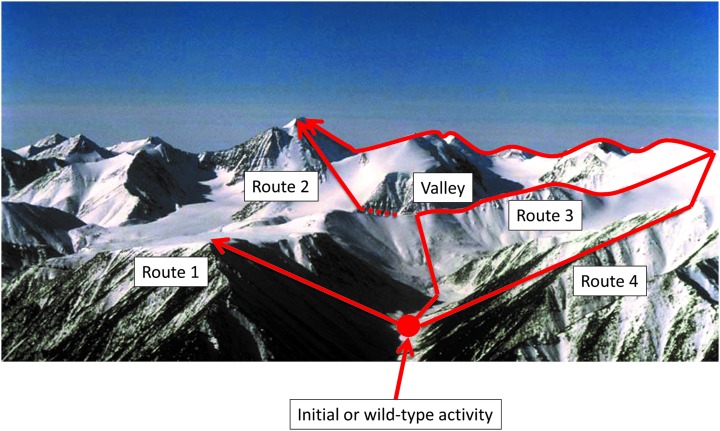
A fitness landscape and its navigation. The initial or wild-type activity denotes the starting point (initialisation) for a directed evolution study (red circle). Accumulation of mutations that increase activity is represented by four routes to different positions in the landscape. Route 1 successfully increases activity through a series of additive mutations, but becomes stuck in a local optimum. Due to the nature of rugged fitness landscapes some of the shorter paths to a maximum possible (global optimum) fitness (activity) can require movement into troughs before navigating a new higher peak (route 2). Alternatively, one can arrive at the global optimum using longer but typically less steep routes without deep valleys (equivalent over flat ground to neutral mutations – routes 3 and 4).

The relationship between sequence and function is often considered in terms of a metaphor in which their evolution is seen as akin to traversing a ‘landscape’,^55^ that may be visualised in the same way as one considers the topology of a natural landscape,^56,57^ with the ‘position’ reflecting the sequence and the desirable function(s) or fitness reflected in the ‘height’ at that position in the landscape (Fig. 5).

Given the enormous numbers for populating sequence space, and the present impossibility of computing or sampling function from sequence alone, it is clear that natural evolution cannot possibly have sampled all possible sequences that might have biological function.^58^ Hence, the strategy of a DE project faces the same questions as those faced in nature: how to navigate sequence space effectively while maintaining at least some function, but introducing sufficient variation that is required to improve that function. For DE there are also the practical considerations: how many variants can be screened (and/or selected for) and analysed with our current capabilities?

The first general point to be made is that most completely random proteins are practically non-functional.^10,56,59–66^ Indeed, many are not even soluble,^67,68^ although they may be evolved to become so.^69^ Keefe and Szostak noted that *ca.* 1 in 10^11^ of random sequences have measurable ATP-binding affinity.^70^ Consistent with this relative sparseness of functional protein space is the fact that even if one does have a starting structure(/function), one typically need not go ‘far’ from such a structure to lose structure quite badly,^71^ albeit that with a ‘density’ of only 1 in 10^11^ proteins being functional this implies that all such functional sequences are connected by trajectories involving changes in only a single amino acid^72^ (and see ref. 58). This is also consistent with the fact that sequence space is vast, and only a tiny fraction of possible sequences tend to be useful and hence selected for by natural evolution. One may note^70,73^ that at least some degree of randomness will be accompanied by some structure,^74,75^ functionality or activity. For proteins, secondary structure is understood to be a strong evolutionary driver,^76^ particularly through the binary-patterning (arrangement of hydrophilic/hydrophobic residues),^64,77–84^ and so is the (somewhat related) packing density.^85–89^ In a certain sense, proteins must at some point have begun their evolution as more or less random sequences.^90^ Indeed “Folded proteins occur frequently in libraries of random amino acid sequences”,^91^ but quite small changes can have significantly negative effects.^92^ Harms and Thornton give a very thoughtful account of evolutionary biochemistry,^4^ recognizing that the “physical architecture {of proteins both} facilitates and constrains their evolution”. This means that it will be hard (but not impossible), especially without plenty of empirical data,^93^ to make predictions about the best trajectories. Fortunately, such data are now beginning to appear.^57,94^ Indeed, the leitmotiv of this review is that understanding such (sequence-structure–activity) landscapes better will assist us considerably in navigating them.

### What is evolving and for what purpose?

In a simplistic way, it is easy to assume that protein sequences are being selected for on the basis of their contribution to the host organism's fitness, without normally having any real knowledge of what is in fact being implied or selected for. However, a profound and interesting point has been made by Keiser *et al.* ^95^ to the effect that once a metabolite has been ‘chosen’ (selected) to be part of a metabolic or biochemical network, proteins are somewhat constrained to evolve as ‘slaves’, to learn to bind and react with the metabolites that exist. Thus, in evolution, the proteins follow the metabolites as much as *vice versa*, making knowledge of ligand binding^96,97^ and affinity^98^ to protein binding sites a matter of primary interest, especially if (as in the DE of biocatalysts) we wish to bind or evolve catalysts for novel (and xenobiotic) small molecule substrates. In DE we largely assume that the experimenter has determined what should be the objective function(s) or fitness(es), and we shall indicate the nature of some of the choices later; notwithstanding, several aspects of DE do tend to differ from those selected by natural evolution (Table 1). Thus, most mutations are pleiotropic *in vivo*,^99,100^ for instance. As DNA sequencing becomes increasingly economical and of higher throughput^101,102^ a greater provenance of sequence data enables a more thorough knowledge of the entire evolutionary landscape to be obtained. In the case of short sequences most^103^ or all^104^ of the entire genotype-fitness landscape may be measured experimentally. We note too (and see later) that there are equivalent issues in the optimization and algorithms of evolutionary computing (*e.g.* ref. 105–107), where strategies such as uniform cross-over,^108^ with no real counterpart in natural or experimental evolution, have been shown to be very effective.

**Table 1:** Some features by which natural evolution, classical DE of biocatalysts, and directed evolution of biocatalysts using synthetic biology differ from each other. Population structures also differ in natural evolution *vs*. DE, but in the various strategies for DE they follow from the imposed selection in ways that are difficult to generalize

Feature	Natural evolution	Classical DE	DE with synthetic biology
Objective function and selection pressure	Unclear; there is only a weak relation of a protein's function with organismal fitness;^117^ *k*_cat_ is not strongly selected for. Although presumably multi-objective, actual selection and fitness are ‘composites’. If there is no redundancy, organisms must retain function during evolution.^58,118^	Typically strong selection weak mutation (rarely was sequencing done so selection was based on fitness only). Can select explicitly for multiple outputs (*e.g. k*_cat_, thermostability).	Much as with classical DE, but diversity maintenance can be much enhanced *via* high-throughput methods of DNA synthesis and sequencing.
Mutation rates	Varies with genome size over orders of magnitude,^119^ but typically (for organisms from bacteria to humans) <10^–8^ per base per generation.^120,121^ Can itself be selected for.^122^	Mutation rates are controlled but often limited to only a few residues per generation, *e.g.* to 1/*L* where *L* is the aa length of the protein; much more can lead to too many stop codons.	Library design schemes that permit stop codons only where required mean that mutation rates can be almost arbitrarily high.
Recombination rates	Very low in most organisms (though must have occurred in cases of ‘horizontal gene transfer’); in some cases almost non-existent.^123^	Could be extremely high in the various schemes of DNA shuffling, including the creation of chimaeras from different parents.	Again it can be as high or low as desired; the experimenter has (statistically) full control.
Randomness of mutation	Although there are ‘hot spots’, mutations in natural evolution are considered to be random and not ‘directed’.^124^	In error-prone PCR, mutations are seen as essentially random. Site-directed methods offer control over mutations at a small number of specified positions.	As much or as little randomness may be introduced as the experimenter desires by using defined mixtures of bases for each codon, *e.g.* NNN or NNK as alternatives to specific subsets such as polar or apolar.
Evolutionary ‘memory’	For individuals (*cf*. populations^125^) there is no ‘memory’ as such, although the sequence reflects the evolutionary ‘trace’ (but not normally the pathway – *cf.* ref. 126 and 127).	Again, there is no real ‘memory’ in the absence of large-scale sequencing, but there is potential for it.^56^	With higher-throughput sequencing we can create an entire map of the landscape as sampled to date, to help guide the informed assessment of which sequences to try next.
Degree of epistasis	It exists, but only when there is a more or less neutral pathway joining the epistatic sites.	It is comparatively hard to detect at low mutation rates.	Potentially epistasis is much more obvious as sites can be mutated pairwise or in more complex designed patterns.
Maintenance of individuals of lower or similar fitness in population	They are soon selected out in a ‘strong selection, weak mutation’ regime; this limits jumps *via* lower fitness, and enforces at least neutral mutations.	It is in the hands of the experimenter, and usually not done when only fitnesses are measured.	Again it is entirely up to the experimenter; diversity may be maintained to trade exploration against exploitation.

**Fig. 6:**
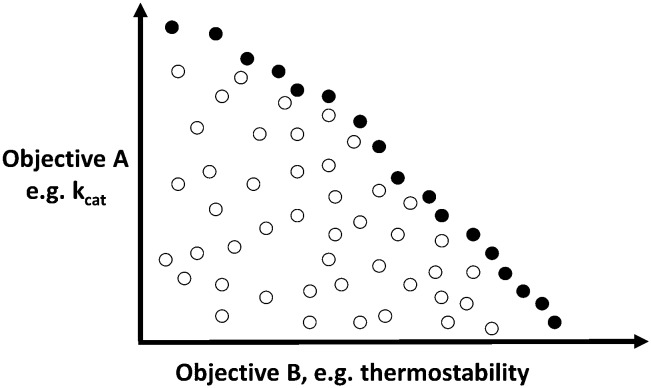
A two-objective optimisation problem, illustrating the non-dominated or Pareto front. In this case we wish to maximise both objectives. Each individual symbol is a candidate solution (*i.e.* protein sequence), with the filled ones denoting an approximation to the Pareto front.

However, in the case of multi-objective optimisation (*e.g.* seeking to optimise two objectives such as both *k*_cat_ and thermostability, or activity *vs.* immunogenicity^109^), there is normally no individual preferred solution that is optimal for all objectives,^110^ but a set of them, known as the Pareto front (Fig. 6), whose members are optimal in at least one objective while not being bettered (not ‘dominated’) in any other property by any other individual. The Pareto front is thus also known as the non-dominated front or ‘set’ of solutions. A variety of algorithms in multi-objective evolutionary optimisation (*e.g.* ref. 111–116) use members of the Pareto front as the choice of which ‘parents’ to use for mutation and recombination in subsequent rounds.

### Protein folds and convergent and divergent evolution

What is certain, given that form follows function, is that natural evolution has selected repeatedly for particular kinds of secondary and tertiary structure ‘domains’ and ‘folds’.^128,129^ It is uncertain as to how many more are ‘common’ and are to be found *via* the methods of structural genomics,^130^ but many have been expertly classified,^131^ *e.g.* in the CATH,^132–134^ SCOP^135–137^ or InterPro^138,139^ databases, and do occur repeatedly.

Given that structural conservation of protein folds can occur for sequences that differ markedly from each other, it is desirable that these analyses are done at the structural (rather than sequence) level (although there is a certain arbitrariness about where one fold ends and another begins^140,141^). Some folds have occurred and been selected *via* divergent evolution (similar sequences with different functions)^142^ and some *via* convergent evolution (different sequences with similar functions).^143,144^ This latter in particular makes the nonlinear mapping of sequence to function extremely difficult, and there are roughly two unrelated sequences for each E.C. (Enzyme Commission classification) number.^145^ As phrased by Ferrada and colleagues,^146^ “two proteins with the same structure and/or function in our data…{have} a median amino acid divergence of no less than 55 percent”. However, normally information is available only for extant molecules but not their history and precise evolutionary path (in contrast to DE). One conclusion might be that conventional means of phylogenetic analysis are not necessarily best placed to assist the processes of directed evolution, and we argue later (because a protein has no real ‘memory’ of its full evolutionary pathway) that modern methods of machine learning that can take into account ensembles of sequences and activities may prove more suitable. However, we shall first look at natural evolution.

### Constraints on globular protein evolution structure in natural evolution

In gross terms, a major constraint on protein evolution is provided by thermodynamics, in that proteins will have a tendency to fold up to a state of minimum free energy.^147–149^ Consequently, the composition of the amino acids has a major influence over protein folding because this means satisfying, so far as is possible, the preference of hydrophilic or polar amino acids to bind to each other and the equivalent tendency of hydrophobic residues to do so.^150–152^ Alteration of residues, especially non-conservatively, often leads to a lowering of thermodynamic folding stability,^153^ which may of course be compensated by changes in other locations. Naturally, at one level proteins need to have a certain stability to function, but they also need to be flexible to effect catalysis. This is coupled to the idea that proteins are marginally stable objects in the face of evolution.^154–159^ Overall, this is equivalent to ‘evolution to the edge of chaos’,^160,161^ a phenomenon recognizing the importance of trading off robustness with evolvability that can also be applied^162,163^ to biochemical networks.^164–170^ Thermostability (see later) may also sometimes (but not always^171–173^) correlate with evolvability.^174,175^

**Fig. 7:**
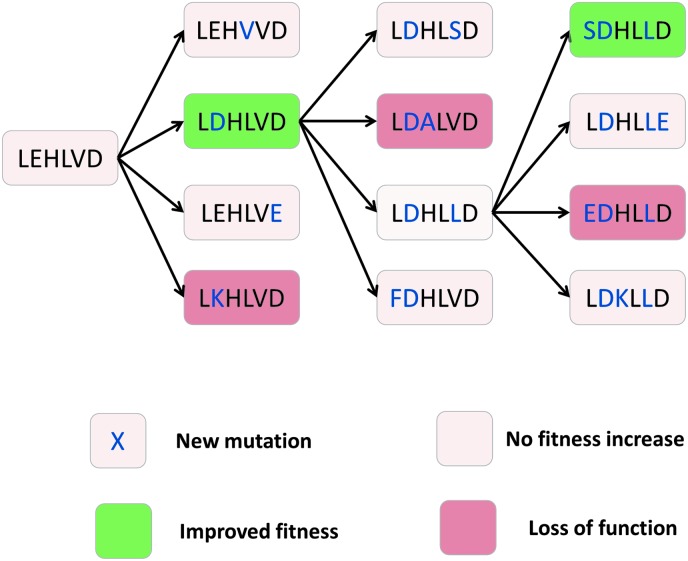
Some evolutionary trajectories of a peptide sequence undergoing mutation. Mutations in the peptide sequence can cause an increase in fitness (*e.g.* enzyme activity, green), loss of fitness (salmon pink) or no change in fitness (grey). Typically, improved fitness mutations are selected for and subjected to further modification and selection. Neutral mutations keep sequences ‘alive’ in the series, and these can often be required for further improvements in fitness, as shown in steps 2 and 3 of this trajectory.

Given the thermodynamic and biophysical^157,176,177^ constraints, that are related to structural contacts, various models (*e.g.* ref. 147 and 178) have been used to predict the distribution of amino acids in known proteins. As regards to specific mechanisms, it has been stated that “solvent accessibility is the primary structural constraint on amino acid substitutions and mutation rates during protein evolution.”,^148^ while “satisfaction of hydrogen bonding potential influences the conservation of polar sidechains”.^179^ Overall, given the tendency in natural evolution for strong selection, it is recognized that a major role is played by neutral mutations^180–182^ or neutral evolution^183–188^ (see Fig. 5 and 7). Gene duplication provides another strategy, allowing redundancy followed by evolution to new functions.^189^

### Coevolution of residues

Thus far, we have possibly implied that residues evolve (*i.e.* are selected for) independently, but that is not the case at all.^190–192^ There can be a variety of reasons for the conservation of sequence (including correlations between ‘distant’ regions^193^), but the importance to structure and function, and functional linkage between them, underlie such correlations.^194–209^ Covariation in natural evolution reflects the fact that, although not close in primary sequence, distal residues can be adjacent in the tertiary structure and may represent an interaction favourable to protein function. Covariation also provides an important computational approach to protein folding more generally (see below).

### The nature, means of analysis and traversal of protein fitness landscapes

Since John Holland's brilliant and pioneering work in the 1970s (reprinted as ref. 210), it has been recognized that one can search large search spaces very effectively using algorithms that have a more or less close analogy to that of natural evolution. Such algorithms are typically known as genetic or evolutionary algorithms (*e.g.* ref. 106 and 211–213, and their implementation is referred to as evolutionary computing.^106,214–216^ The algorithms can be classified according to whether one knows only the fitnesses (phenotypes) of the population or also the genotypes (sequences).^107^

Since we cannot review the very large literature, essentially amounting to that of the whole of molecular protein evolution, on the nature of (natural) protein landscapes, we shall therefore seek to concentrate on a few areas where an improved understanding of the nature of the landscape may reasonably be expected to help us traverse it. Importantly, even for single objectives or fitnesses, a number of important concepts of ruggedness, additivity, promiscuity and epistasis are inextricably intertwined; they become more so where multiple and often incommensurate objectives are considered.

#### Additivity

Additivity implies simple continuing fixing of improved mutations,^217–220^ and follows from a model in which selection in natural evolution quite badly disfavours lower fitnesses,^221^ a circumstance known from Gillespie^222,223^ as ‘strong selection, weak mutation’ (SSWM, see also ref. 224–229). For small changes (close to neutral in a fitness or free energy sense), additivity may indeed be observed,^230,231^ and has been exploited extensively in DE.^232–236^ If additivity alone were true, however (and thus there is no epistasis for a given protein at all) then a rapid strategy for DE would be to synthesise all 20*L* amino acid variants at each position (of a starting protein of length *L*) and pick the best amino acid at each position. However, the very existence of convergent and divergent evolution implies that landscapes are rugged^237^ (and hence epistatic), so at the very least additivity and epistasis must coexist.^236,238^

#### Epistasis

The term ‘epistasis’ in DE covers a concept in which the ‘best’ amino acid at a given position depends on the amino acid at one or more other positions. In fact, we believe that one should start with an assumption of rather strong epistasis,^238–248^ as did Wright.^55^ Indeed the rugged fitness landscape is itself a necessary reflection of epistasis and *vice versa*. Thus, epistasis may be both cryptic and pervasive,^249^ the demonstrable coevolution goes hand in hand with epistasis, and “to understand evolution and selection in proteins, knowledge of coevolution and structural change must be integrated”.^250^

#### Promiscuity

The concept of enzyme promiscuity mainly implies that some enzymes may bind, or catalyse reactions with, more than one substrate, and this is inextricably linked to how one can traverse evolutionary landscapes.^251–270^ It clearly bears strongly on how we might seek to effect the directed evolution of biocatalysts.

### NK landscapes as models for sequence-activity landscapes

A very important class of conceptual (and tunable) landscapes are the so-called NK landscapes devised by Kauffman^161,271^ and developed by many other workers (*e.g.* ref. 220, 221, 237 and 272–278). The ‘ruggedness’ of a given landscape is a slightly elusive concept,^279^ but can be conceptualized^56,220^ in a manner that implies that for a smooth landscape (like Mt Fuji^280,281^) fitness and distance tend to be correlated, while for a very ‘rugged’ landscape the correlation is much weaker (since as one moves away from a starting sequence one may pass through many peaks and troughs of fitness). In NK landscapes, *K* is the parameter that tunes the extent of ruggedness, and it is possible to seek landscapes whose ruggedness can be approximated by a particular value of *K*, since one of the attractions of NK is that they can reproduce (in a statistical sense) any kind of landscape.^282^ Indeed, we can use the comparatively sparse data presently available to determine that experimental sequence-fitness landscapes reflect NK landscapes that are fairly considerably (but not pathologically) rugged,^23,57,104,241,251,274,276,283^ and that there is likely to be one or more optimal mutation rates that themselves depend on the ruggedness (see later). Note too that the landscapes for individual proteins, as discussed here, are necessarily more rugged than are those of pathways or organisms, due to the more profound structural constraints in the former.^57,157^ (Parenthetically, NK-type landscapes and the evolutionary metaphor have also proved useful in a variety of other ‘complex’ spheres, such as business, innovation and economics (*e.g.* ref. 278 and 284–295, though a disattraction of NK landscapes in evolutionary biology itself is that they do not obey evolutionary rules.^224^)

## Experimental directed protein evolution

A number of excellent books and review articles have been devoted to DE, and a sampling with a focus on biocatalysis includes.^296–334^ As indicated above, DE begins with a population that we hope contains at least one member that displays some kind of activity of interest, and progresses through multiple rounds of mutation, selection and analysis (as per the steps in Fig. 4).

## Initialisation; the first generation

During the preliminary design of a DE project the main objective and required fitness criteria must be defined and these criteria influence the experimental design and screening strategy.

We consider in this review that a typical scenario is that one has a particular substrate or substrate class in mind, as well as the chemical reaction type (oxidation, hydroxylation, amination and so on) that one wishes to catalyse. If any activity at all can be detected then this can be a starting point. In some cases one does not know where to start at all because there are no proteins known either to catalyse a relevant reaction or to bind the substrate of interest. For pharmaceutical intermediates, it can still be useful to look for reactions involving metabolites, as most drugs do bear significant structural similarities to known metabolites,^335,336^ and it is possible to look for reactions involving the latter. A very useful starting point may be the structure-function linkage database ; http://sfld.rbvi.ucsf.edu/django/.^337^ There are also ‘hub’ sequences that can provide useful starting points,^338^ while Verma,^330^ Nov^339^ and Zaugg^340^ list various computational approaches. If one has a structure in the form of a PDB file one can try HotSpotWizard ; http://loschmidt.chemi.muni.cz/hotspotwizard/.^341^ Analysing the diversity of known enzyme sequences is also a very sensible strategy.^342,343^ Nowadays, an increasing trend is to seek relevant diversity, aligned using tools such as Clustal Omega,^344,345^ MUSCLE,^346^ PROMALS,^347,348^ or other methods based on polypharmacology,^141,349,350^ that one may hope contains enzymes capable of effecting the desired reaction. Another strategy is to select DNA from environments that have been exposed to the substrate of interest, using the methods of functional metagenomics.^351,352^ More commonly, however, one does have a very poor protein (clone) with at least some measurable activity, and the aim is to evolve this into a much more active variant.

**Fig. 8:**
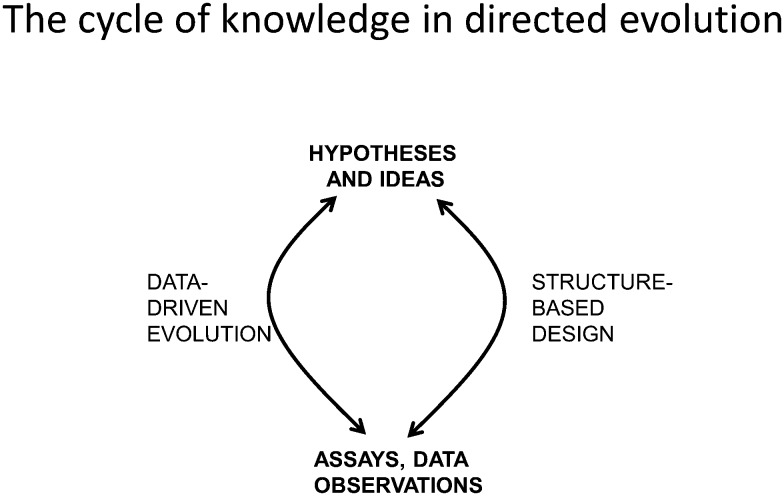
The ‘cycle of knowledge’ in modern directed evolution. Both structure-based design and a more empirical data-driven approach can contribute to the evolution of a protein with improved properties, in a series of iterative cycles.

In general, scientific advance is seen in a Popperian view (see *e.g.* ref. 353–357) as an iterative series of ‘conjectures’ and ‘refutations’ by which the search for scientific truth is ‘narrowed’ by finding what is not true (may be falsified) *via* predictions based on hypothetico-deductive reasoning and their anticipated and experimental outcomes. However, Popper was purposely coy about where hypotheses actually came from, and we prefer a variant^358–362^ (see also ref. 363 and 364) that recognises the equal contribution of a more empirical ‘data-driven’ arc to the ‘cycle of knowledge’ (Fig. 8).

In a similar vein, many commentators (*e.g.* ref. 365–368) consider the best strategy for both the starting population and the subsequent steps to be a judicious blend between the more empirical approaches of (semi-)directed evolution and strategies more formally based on attempts to design^369^ (somewhat in the absence of fully established principles) sequences or structures based on what is known of molecular interactions. We concur with this, since at the present time it is simply not possible to design enzymes with high activities *de novo* (from scratch, or from sequence alone), despite progress in simple 4-helix-bundle and related ‘maquettes’.^370–373^ David Baker, probably the leading expert in protein design, considers that design is still incapable of predicting active enzymes even when the chemistry and active sites appear good.^374,375^ Several reviews attest to this,^329,376–379^ but crowdsourcing approaches have been shown to help,^380^ and computational design (and see below) certainly beats random sequences.^381^ Overall, the fairest comment is probably that we can benefit from design for binding, specificity and active site modelling, but that for improving *k*_cat_ we need the more empirical methods of DE, especially (see below) of residues distant from the active site.

### Scaffolds

Because natural evolution has selected for a variety of motifs that have been shown in general terms to admit a wide range of possible enzyme activities, a number of approaches have exploited these motifs or ‘scaffolds’.^382^ Triose phosphate isomerase (TIM) has proved a popular enzyme since the pioneering work of Albery and Knowles^383^ and more recent work on TIM energetics,^384^ and TIM (βα)_8_ barrels can be found in 5 of 6 EC classes.^146^ TIM and many (but not all) such natural enzymes are most active as dimers,^385,386^ caused by a tight interaction of 32 residues of each subunit in the wild type, though functional monomers can be created.^387,388^

Thus, (βα)_8_ barrel enzymes^389–402^ have proven particularly attractive as scaffolds for DE.^403–407^ Some use or need cofactors like PLP, FMN, *etc.*,^392,408^ and their folding mechanisms are to some degree known.^385,409–411^ We note, however, that virtual screening of substrates against these^412^ has shown a relative lack of effectiveness of consensus design because of the importance of correlations (*i.e.* epistasis).^386^

α/β and (α/β)_2_ barrels have also been favoured as scaffolds,^395,413–417^ while attempts at automated scaffold selection can also be found.^374,418,419^ A very interesting suggestion^420^ is that the polarity of a fold may determine its evolvability.

Although not focused on biocatalysis, other scaffolds such as lipocalins^421–426^ and affibodies^427–437^ have proved useful for combinatorial biosynthesis and directed evolution.

### Computational protein design

While computational protein design completely from scratch (*in silico*) is not presently seen as reasonable, probably (as we stress later) because we cannot yet use it to predict dynamics effectively, significant progress continues to be made in a number of areas,^373,438–456^ including ‘fold to function’,^457^ combinatorial design,^458^ and a maximum likelihood framework for protein design.^459^ Notable examples include a metalloenzyme for organophosphate hydrolysis,^460,461^ aldolase^462,463^ and others.^464–468^ Theozymes^469–472^ (theoretical catalysts, constructed by computing the optimal geometry for transition state stabilization by model functional) groups represent another approach.

Arguably the most advanced strategies for protein design and manipulation *in silico* are Rosetta^374,473–483^ and RosettaBackrub,^484,485^ while more ‘bottom-up’ approaches, based on some of the ideas of synthetic biology, are beginning to appear.^486–492^ It is an easy prediction that developments in synthetic biology will have highly beneficial effects on *de novo* design, and *vice versa*.

### Docking

If one is to find an enzyme that catalyses a reaction, one might hope to be able to predict that it can at least bind that substrate using the methods of *in silico* docking.^493^ To date, methods based on Autodock,^494–499^ APoC,^500^ Glide^501–503^ or other programs^504–511^ have been proposed, but this strategy is not yet considered mainstream for the DE of a first generation of biocatalysts (and indeed is subject to considerable uncertainty^512^). Our experience is that one must have considerable knowledge of the approximate answer (the binding site or pocket) before one tries these methods for DE of a biocatalyst.

Having chosen a member (or a population) as a starting point, the next step in any DE program is the important one of diversity creation. Indeed, the means of creating and exploiting suitable libraries that focus on appropriate parts of the protein landscape lies at the heart of any intelligent search method.^513^

## Diversity creation and library design

**Fig. 9:**
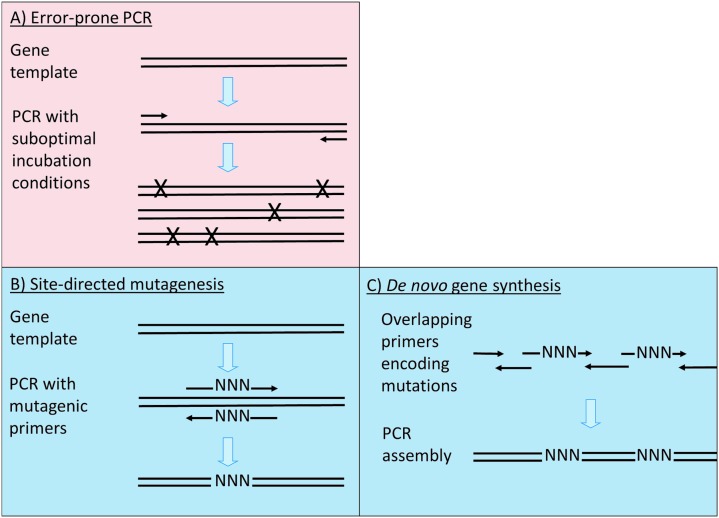
Overview of the different mutagenesis strategies commonly employed to create variant protein libraries. Random methods (pink background) can create the greatest diversity of sequences in an uncontrolled manner. Mutations during error-prone PCR (A) are typically introduced by a polymerase amplifying sequences imperfectly (by being used under non-optimal conditions). In contrast, directed mutagenesis methods (blue background) introduce mutations at defined positions and with a controlled outcome. Site-directed mutagenesis (B) introduces a mutation, encoded by oligonucleotides, onto a template gene sequence in a plasmid. However, gene synthesis (C) can encode mutations on the oligonucleotides used to synthesise the sequence *de novo*, hence multiple mutations can be introduced simultaneously. X = random mutation, N = controlled mutation. →= PCR primer.

**Fig. 10:**
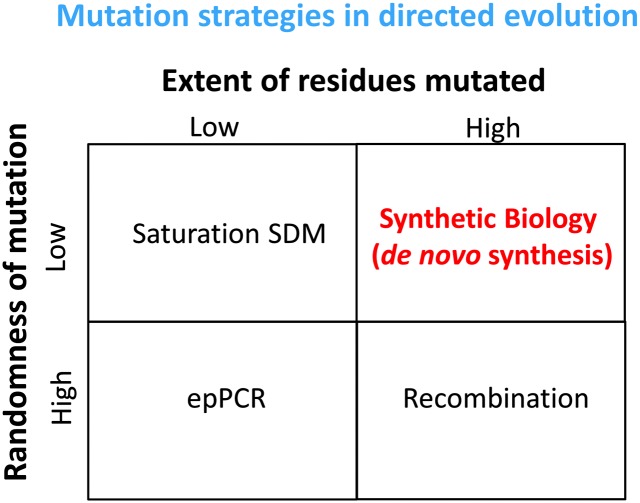
A Boston matrix of the different strategies for variant libraries. Methods are identified in terms of the randomness of the mutations they create and the number of residues that can be targeted.

A diversity of sequences can be created in many ways,^514^ but mutation or recombination methods are most commonly used in DE. Some are purely empirical and statistical (*e.g. N* mutations per sequence), while others are more focused to a specific part of the sequence (Fig. 9). Strategies may also be discriminated in terms of the degree of randomness of the changes and their extensiveness (Fig. 10). Two useful reviews include^515^ and,^516^ while others^334,517–519^ cover computational approaches. A DE library creation bibliography is maintained at ; http://openwetware.org/wiki/Reviews:Directed_evolution/Library_construction/bibliography.

### Effect of mutation rates, implying that higher can be better

In classical evolutionary computing, the recognition that most mutations were or are deleterious meant that mutation rates were kept low. If only one in 10^3^ sequences is an improvement when the mutation rate is 1/*L* per position (*L* being the length of the string), then (in the absence of epistasis) only 1 in 10^6^ is at 2/*L*. (Of course 1/*L* is far greater than the mutation rates common in natural evolution, which scales inversely with genome size,^119^ may depend on cell–cell interactions,^520^ and is normally below 10^–8^ per base per generation for organisms from bacteria to humans.^119–121^) This logic is persuasive but limited, since it takes into account only the frequency but not the quality of the improvement (and as mentioned essentially does not consider epistasis). Indeed there is evidence that higher mutation rates are favoured both *in silico* ^220,521–524^ and experimentally.^525–528^ This is especially the case for directed mutagenesis methods (especially those of synthetic biology), where stop codons can be avoided completely. We first discuss the more classical methods.

### Random mutagenesis methods

Error-prone PCR (epPCR) is probably the most commonly used method for introducing random mutations. PCR amplification using *Taq* polymerase is performed under suboptimal conditions by altering the components of the reaction (in particular polymerase concentration, MgCl_2_ and dNTP concentration, or supplementation with MnCl_2_ (ref. 529)) or cycling conditions (increased extension times).^530^ Although epPCR is the simplest to implement and most commonly used method for library creation, it is limited by its failure to access all possible amino acid changes with just one mutation,^339,531–533^ a strong bias towards transition mutations (AT to GC mutations),^531^ and an aversion to consecutive nucleotide mutations.^532,534^

Refinement of these methods has allowed greater control over the mutation bias, rate of mutations^530,535–537^ and the development of alternative methodologies like Mutagenic Plasmid Amplification,^538^ replication,^539^ error-prone rolling circle^540^ and indel^541–543^ mutagenesis. Typically, for reasons indicated above, the epPCR mutation rate is tuned to produce a small number of mutations per gene copy (although orthogonal replication *in vivo* may improve this^544^), since entirely random epPCR produces multiple stop codons (3 in every 64 mutations) and a large proportion of non-functional, truncated or insoluble proteins.^545^ The library size also dictates that a large number of mutants must be screened to test for all possibilities, which may also be impractical depending on the screening strategy available. While random methods for library design can be successful, intelligent searching of the sequence space, as per the title of this review, does not include *purely* random methods.^546^ In particular, these methods do not allow information about which parts of the sequence have been mutated or whether all possible mutations for a particular region of interest have been screened.

### Site-directed mutagenesis to target specific residues

Since the combinatorial explosion means that one cannot try every amino acid at every residue, one obvious approach is to restrict the number of target residues (in the following sections we will discuss why we do not think this is the best strategy for making faster biocatalysts). Indeed, mutagenesis directed at specific residues, usually referred to as site-directed mutagenesis,^547,548^ dates from the origins of modern protein engineering itself.^549^

**Fig. 11:**
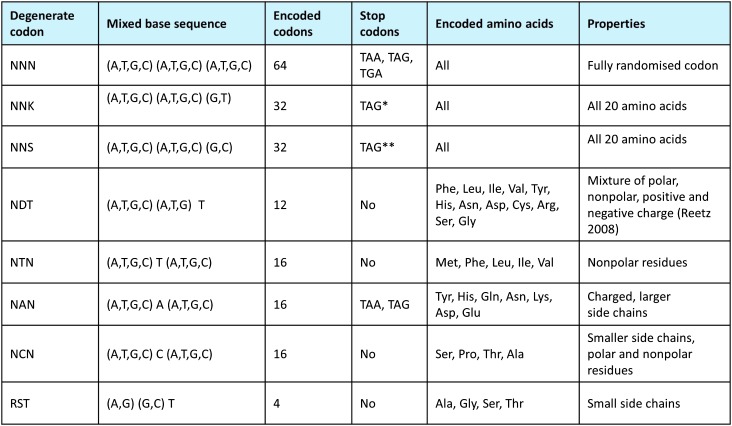
Examples of some of the common degenerate codons used in DE studies. A codon containing specific mixed bases is used to encode a particular set of amino acids, ranging from all twenty amino acids (NNN or NNK) to those with particular properties. Hence, choice of degenerate codons to use depends on the design and objective of the study. In the IUPAC terminology^590^ K = G/T, M = A/C, R = A/G, S = C/G, W = A/T, Y = C/T, B = C/G/T, D = A/G/T, H = A/C/T, V = A/C/G, N = A/C/G/T. (*Typically with low codon usage; suppressor mutation may be used to block it. **Typically with low codon usage, especially in yeast; suppressor mutation may be used to block it).

In site-directed mutagenesis, an oligonucleotide encoding the desired mutation is designed with flanking sequences either side that are complementary to the target sequence and these direct its binding to the desired sequence on a template. This oligomer is used as a PCR primer to amplify the template sequence, hence all amplicons encode the desired mutation. This control over the mutation enables particular types of mutation to be made by using mixed base codons, *i.e.* codons that contain a mixture of bases at a specified position (*e.g.* N denotes an equal mixture of A, T, G or C at a single position). Fig. 11 shows a compilation of the more common types of mixed codons used. These range from those capable of encoding all 20 amino acids (*e.g.* NNK) to a small subset of residues with a particular physicochemical property (*e.g.* NTN for nonpolar residues only).

The most common method (QuikChange and derivatives thereof) uses mutagenic oligonucleotides complementary to both strands of a target sequence, which are used as primers for a PCR amplification of the plasmid encoding the gene. Following DpnI digestion of the template, the PCR product is transformed into *E. coli* and the nicked plasmid is repaired *in vivo*.^550,551^ Despite its popularity, QuikChange is somewhat limited by aspects like primer design and efficiency, and a variety of derivatives have been published that improve upon the original method.^552,553^

Given that site-directed mutagenesis provides a way of mutating a small number of residues with high levels of accuracy, several approaches have been developed to identify possible positions to target to increase the hit rate and success. Combinatorial alanine scanning^554,555^ is well known, while other flavours include the Mutagenesis Assistant Program,^531,556^ and the semi-rational CASTing and B-FIT approaches^323,557^ that employ a Mutagenic Plasmid Amplification method.^558^

In addition to these more conventional methods, new approaches are continually being developed to improve efficiency and to reduce the number of steps in the workflow, for example Mutagenic Oligonucleotide-Directed PCR Amplification (MOD-PCR),^559^ Overlap Extension PCR (OE-PCR),^560–564^ Sequence Saturation Mutagenesis,^565–571^ Iterative Saturation Mutagenesis,^557,572–579^ and a variety of transposon-based methods.^580–583^ However, a common issue with site-directed mutagenesis methods is the large number of steps involved and the limited number of positions that can be efficiently targeted at a time. The ability to mutate residues in multiple positions in a sequence is of particular interest as this can be used to address the question of combinatorial mutations simultaneously. Hence, methods like those by Liu *et al.*,^584^ Seyfang *et al.*,^585^ Fushan *et al.* ^586^ and Kegler-Ebo *et al.* ^587^ are important developments in mutagenesis strategies. Rational approaches have been reviewed,^588^ including from the perspective of the necessary library size.^589^ As a result, there is significant interest in the development of novel methodologies that can address these issues to produce accurate variant libraries, with larger numbers of simultaneous mutations in an economical workflow.

### Optimising nucleotide substitutions

Following the selection of residues to target for mutation an important choice is the type of mutation to create. This choice is not obvious but determines the type of mutations that are made and the level of screening required. The experimenter needs to consider the nature of the mutations that they want to introduce for each position and this relates to the objective of the study. Using the common mixed base IUPAC terminology^590^ (Fig. 11) there are a large number of codons that can be chosen, ranging from those encoding all 20 amino acids (the NNK or NNS codons), to a particular characteristic (*e.g.* NTN encodes just nonpolar residues^64^) and a limited number of defined residues (GAN encoding just aspartate or glutamate). Importantly, choosing to use these specified mixed base codons in mutagenesis can reduce the possibility of premature stop codons and increase the chance of creating functional variants. For example, if a wild-type sequence encodes a nonpolar residue at a particular position then the number of functional variants is likely to be higher if the nonpolar codon NTN is used, encoding what are conserved substitutions, compared to encoding all possible residues with the NNK codon.^591,592^

Indeed, it is known to be better to search a large library sparsely than a small library thoroughly.^593^ Thus, a general strategy that seeks to move the trade-off between numbers of changes and numbers of clones to be assessed recognizes that one can design libraries that cover different general amino acid properties (such as charged, hydrophobic) while not encoding all 20 amino acids, thereby reducing (somewhat) the size of the search space. These are known as reduced library designs (see Fig. 11).

### Reduced library designs

One limitation with the use of single degenerate codons is that for some sequences not all amino acids are equally represented and sometimes rare codons or stop codons are encoded. To circumvent this issue “small-intelligent” or “smart” libraries have been developed to provide equal frequency of each amino acid without bias.^594^ Using a mixture of oligonucleotides, Kille *et al.* ^595^ created a restricted library with three codons NDT, VHG and TGG that encode 12, 9 and 1 codon, respectively. Together these encode 22 codons for all 20 amino acids in equal frequency, which provides good coverage of possible mutations but reduces the screening effort required to cover the sequence space. Alternative methods with the same objective include the MAX randomisation strategy^596^ and using ratios of different degenerate codons designed by software (DC-Analyzer^597^). Alternatively, the use of a reduced amino acid alphabet can also search a relevant sequence space whilst reducing the screening effort further. For example, the NDT codon encodes 12 amino acids of different physicochemical properties without encoding stop codons and has been shown to increase the number of positive hits (*versus* full randomization) in directed evolution studies.^324^ Overall, a considerable number of such strategies have been used (*e.g.* ref. 64, 67, 68, 81, 82, 324, 513, 556, 592 and 596–603).

The opposite strategy to reduced library designs is to increase them by modifying the genetic code. While one may think that there is enough potential in the very large search spaces using just 20 amino acids, such approaches have led to some exceptionally elegant work that bears description.

### Non-canonical amino acid incorporation

If the existing protein synthetic machinery of the host cell is able to recognise a novel amino acid, it is possible to take an auxotroph and add the non-canonical amino acid (NCAA)^604^ that is thereby incorporated non-selectively. If one wishes to have site specificity, there are two main ways to increase the number of amino acids that can be incorporated into proteins.^605^ First, the specificity of a tRNA molecule (*e.g.* one encoding a stop codon) can be modified to accommodate non-canonical amino acids; in this way, the use of the relevant codon can introduce an NCAA at the specified position.^606,607^ Using this method, eight NCAAs were incorporated into the active site of nitroreductase (NTR, at Phe124) and screened for activity. One Phe analogue, *p*-nitrophenylalanine (*p*NF), exhibited more than a two-fold increase in activity over the best mutant containing a natural amino acid substitution (P124K), showing that NCAAs can produce higher enzyme activity than is possible with natural amino acids.^608^

The other, considerably more radical and potentially ground-breaking, is effectively to evolve the genetic code and other apparatus such that instead of recognising triplets a subset of mRNAs and the relevant translational machinery can recognise and decode quadruplets.^609–619^ To date, some 100 such NCAAs have been incorporated. However, the incorporation of NCAAs can often impact negatively on protein folding and thermostability, an issue that can be addressed through further rounds of directed evolution.^620^

### Recombination

**Fig. 12:**
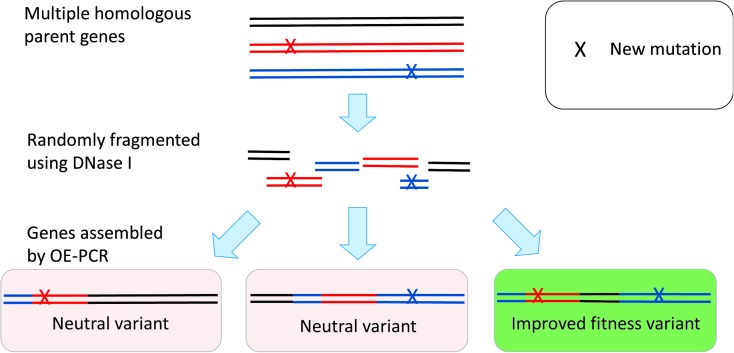
The traditional recombination method for diversity creation. Recombination requires a sample of different variants of a gene (parents), which can be derived from a family of homologous genes or generated by random mutagenesis methods. The random fragmentation of these genes (using DNase I or other method) cleaves them into small constituent parts. Importantly, as the parental genes are all homologous, the fragments overlap in sequence thus allowing them to be reassembled by overlap extension PCR (OE-PCR) producing products that encode a random mixture of the parental genes. A key advantage of recombination methods is the improved ability to create combinatorial mutations. This is illustrated using two mutations (present in two different parental sequences) that when recombined separately produce no fitness improvement, but when combined together produce a variant with improved fitness.

In contrast to the mutagenesis methods of library creation outlined above, but entirely consistent with our knowledge from strategies used in evolutionary computing (*e.g.* ref. 106), recombination is an alternative (or complementary) and effective strategy for DE (Fig. 12). Recombination techniques offer several advantages that reflect aspects of natural evolution that differ from random mutagenesis methods, not least because such changes can be combinatorial and hence able to search more areas of the sequence space in a given experiment. Recombination for the purposes of DE was popularized by Stemmer and his colleagues under the term ‘DNA shuffling’.^621–625^ This used a pool of parental genes with point mutations that were randomly fragmented by DNAseI and then reassembled using OE-PCR. Since then, a variety of further methods have been developed using different fragmentation and assembly protocols.^626–629^ Parental genes for DNA shuffling can be generated by random mutagenesis (epPCR) or from homologous gene families; such chimaeras may be particularly effective.^630–633^

Despite its advantages for searching wider sequence space, however, such recombination does not yield chimaeric proteins with balanced mutation distribution. Bias occurs in crossover regions of high sequence identity because the assembly of these sequences is more favourable during OE-PCR.^634,635^ As a result, this reduces the diversity of sequences in the variant library. Alternative methods like SCRATCHY^636,637^ generate chimaeras from genes of low sequence homology and so may help to reduce the extent of bias at the crossover points.

In addition to these more traditional methods of DNA shuffling, a number of variations have been developed (often with a penchant for a quirky acronym), such as Iterative Truncation for the Creation of HYbrid enzymes (ITCHY^638,639^), RAndom CHImeragenesis on Transient Templates (RACHITT),^640^ Recombined Extension on Truncated Templates (RETT),^641^ One-pot Simple methodology for CAssette Randomization and Recombination) OSCARR,^642,643^ DNA shuffling Frame shuffling,^644^ Synthetic shuffling,^645^ Degenerate Oligonucleotide Gene Shuffling (DOGS),^646^ USERec,^647,648^ SCOPE^649–651^ and Incorporation of Synthetic Oligos duRing gene shuffling (ISOR).^652,653^ Other methods of recombination that have been used for the improvement of proteins include the Protamax approach,^654^ DNA assembler,^655,656^ homologous recombination *in vitro* ^657^ and Recombineering (*e.g.* ref. 658 and 659). Circular permutation, in which the beginning and end of a protein are effectively recombined in different places, provides a (perhaps surprisingly) effective strategy.^17,660–667^

There has long been a recognition that the better kind of chimaeragenesis strategies are those that maintain major structural elements,^668,669^ by ensuring that crossover occurs mainly or solely in what are seen as structurally the most ‘suitable’ locations. This is the basis of the OPTCOMB,^670,671^ RASPP,^672^ SCHEMA (*e.g.* ref. 673–683) and other types of approach.^109,684–691^

Thus, in the directed evolution of a cytochrome P450, Otey *et al.* ^674^ utilized the SCHEMA algorithm to approximate the effect of recombination with different parent P450s on the protein structure. SCHEMA provided a prediction of preferred positions for crossovers, which enabled the creation of a mutant with a 40-fold higher peroxidase activity.^673,678^ Similarly, the recombination of stabilizing fragments was also able to increase the thermostability of P450s using the same approach.^692^

### Cell-free synthesis

Although the majority of the mutations and recombinations described above have been performed *in vitro*, the actual expression of the proteins themselves, and the analysis of their functionalities, is usually done *in vivo*. However, we should mention a series of purely *in vitro* strategies that have also been used to identify good sequences when coupled to suitable *in vitro* translation systems with functional assays.^693–700^

## Synthetic biology for directed evolution

With the recent improvements in DNA synthesis technology and reducing costs it is becoming increasingly feasible to synthesise sequences on a large scale. The most widely used methods for DNA synthesis continue to be short single-stranded oligodeoxyribonucleotides (typically 10–100 nt in length, often abbreviated to oligonucleotides or oligos) using phosphoramidite chemistry,^701,702^ although syntheses from microarrays have particular promise.^546,703–708^ Following synthesis, these oligonucleotides are assembled into larger constructs using enzymatic methods.

Hence, the foundation of synthetic biology is based on the ability to design and assemble novel biological systems ‘from the ground up’, *i.e.* synthetically at the DNA level.^709–713^ As a result, gene synthesis and genome assembly methods have been developed to create novel sequences of several kilobases in length.^714^ In particular, Gibson *et al.* recently assembled sections of the *Mycoplasma genitalium* genome (each 136 to 166 kb) using overlapping synthesised oligonucleotides.^5,6^

These developments in DNA synthesis technology (and lowered cost) can greatly benefit directed evolution studies. In particular, gene synthesis using overlapping oligonucleotides presents a particularly promising method for introducing controlled mutations into a gene sequence. As these methods assemble the gene *de novo*, multiple mutations at different positions in the gene can be introduced simultaneously in a single workflow, decreasing the need for iterative rounds of mutagenesis.

In this process, oligonucleotide sequences are designed to be overlapping and span the length of the gene of interest, following synthesis they are assembled by either PCR-based^715,716^ or ligation-based^717–720^ methods. Variant libraries can be created using this process by encoding mixed base codons on the oligonucleotides and at multiple positions if required.^721^ However, a limitation of the conventional gene synthesis procedure is the inherent error rate (primarily single base inserts or deletions),^722,723^ which arises from errors in the phosphoramidite synthesis of the oligonucleotides. As a result, clones encoding the desired sequence must be verified by DNA sequencing and an error-correction procedure is often required. Several error-correction methods are used, including site-directed mutagenesis,^724^ mismatch binding proteins^725^ and mismatch cleaving endonucleases.^726,727^ Of these, mismatch endonucleases are the most commonly used, and they are amenable to high throughput and automation.

### SpeedyGenes and GeneGenie: tools for synthetic biology applied to the directed evolution of biocatalysts

Mismatch endonucleases recognise and cleave heteroduplexes in a DNA sequence. Consequently, they can be used as an effective method for the removal of errors during gene synthesis. However, when using mixed-base codons in directed evolution this is problematic, as these mixed sequences will form heteroduplexes and so will be heavily cleaved, thus preventing assembly of the required full-length sequence. Hence, we have developed an improved gene synthesis method, SpeedyGenes, which both improves the accurate synthesis of larger genes and can also accommodate mixed-base codon sequences.^728^ SpeedyGenes integrates a mismatch endonuclease step to cleave mismatched bases and, anticipating complete digestion of the mixed-base sequences, then restores these mixed base sequences by reintroducing the oligonucleotides encoding the mutation back into the PCR (“spiking in”) to allow the full length, error corrected gene to be synthesised. Importantly, multiple variant codons can be encoded at different positions of the gene simultaneously, enabling greater search of the sequence space through combinatorial mutations. This was illustrated^728^ by the synthesis of a monoamine oxidase (MAO-N) with three contiguous mixed-base codons mutated at two different positions in the gene. The known structure of MAO-N showed that the side chains of these residues were known to interact, hence these libraries could be screened for combinatorial coevolutionary mutations.

**Fig. 13:**
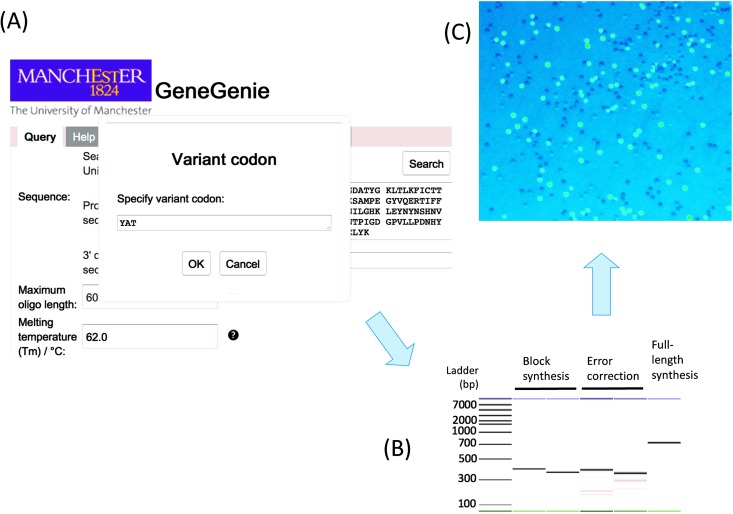
GeneGenie and SpeedyGenes: synthetic biology tools for the purposes of directed evolution. The integration of computational design and accurate gene synthesis methodology provide a strong platform that can be utilised for directed evolution. As an example, the design, synthesis and screening of a small library of EGFP variants is shown. Mixed base codons are used to encode the green and blue variants of EGFP in a single library. (A) GeneGenie (www.gene-genie.org/) designs overlapping oligonucleotides for a given protein together with any specific mixed base codon (here YAT denoting C/T,A,T). (B) SpeedyGenes assembles the gene sequence using these oligonucleotides, accurately (using error correction) producing variant libraries with the desired mutations. (C) Direct expression (no pre-selection) of the library in *E. coli* yielded colonies with the desired mutations (green or blue fluorescence).

As with most synthetic biology methods, the use of sequence design *in silico* is crucial to the successful synthesis *in vitro*. In the case of SpeedyGenes, a parallel, online software design tool, GeneGenie, was developed to automate the design of DNA sequences and the desired variant library.^729^ By calculating the melting temperature (*T*_m_) of the overlapping sequences, and minimising the potential mis-annealing of oligomers, GeneGenie greatly improves the success rate of assembly by PCR *in vitro*. In addition, codons are selected according to the codon usage of the expression host organism, and cloning sequences can be encoded *ab initio* to facilitate downstream cloning. Importantly, any mixed base codon can be added to incorporate into the designed sequence, hence automating the design of the variant library. As an example, a limited library of enhanced green fluorescent protein (EGFP) were designed to encode two variant codons (YAT at Y66 and TWT at Y145), the product of which would encode a limited variant library of green and blue variants of EGFP^728^ (Fig. 13).

## Genetic selection and screening

An important aspect of any experiment exploiting directed evolution for the development of improved biocatalysts is how one determines which of the many millions (or more) of the different clones that are created is worth testing further and/or retaining for subsequent generations. If it is possible to include a (genetic) selection step prior to any screening, this is always likely to prove valuable.^303,730–732^

### Genetic selection

**Fig. 14:**
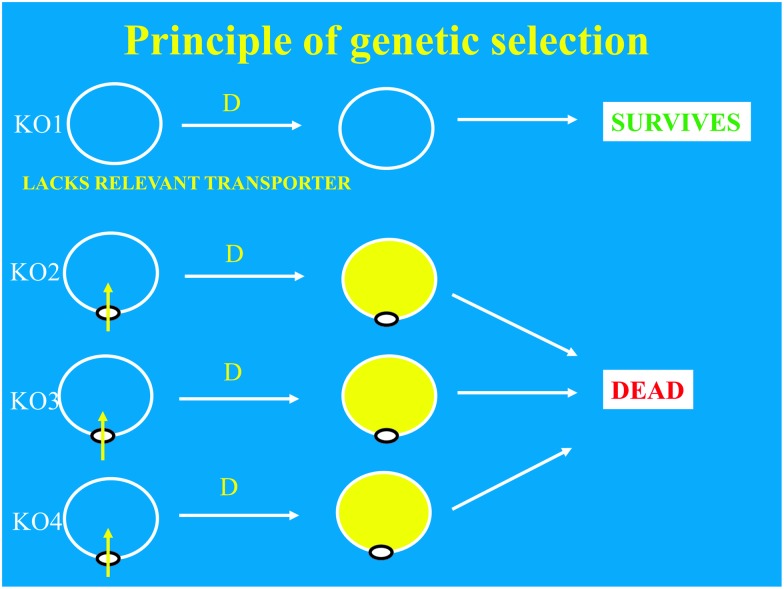
The principle of genetic selection, here illustrated with a transporter gene knockout mutant in competition with others^749^ that does not take up toxic levels of an otherwise cytotoxic drug D.

Most strategies for selection are unique to the protein of interest, and hence need to be designed empirically. Generally, this entails selection of a clone containing a desirable protein because it leads the cell to have a higher fitness.^599,733^ Examples including those based on enantioselectivity,^734,735^ substrate utilisation,^736^ chemical complementation,^737,738^ riboswitches,^739–743^ and counter-selection^744^ can be given. An ideal is when the selection rescues cells from a toxic insult that would otherwise kill them^745^ (see Fig. 14) or repairs a growth defect^746–748^). Two such examples^749,750^ of genetic selection are based on transporter engineering. However, most of the time it is quite difficult to develop such a genetic selection assay, so one must resort to screening.

## Screening

Microtitre plates are the standard in biomolecular screening, and this is no different in DE.^751^ Herein, clones are seeded such that one clone per well is cultured, the substrates added, and the activity or products screened, primarily using chromogenic or fluorogenic substrates. This said, flow cytometry and fluorescence-activated cell sorting (FACS) have the benefit of much higher throughputs and have been widely applied (*e.g.* ref. 415 and 752–790) (and see below for microchannels and picodroplets). 2D arrays using immobilized proteins may also be used.^791,792^ However, not all products of interest are fluorescent, and these therefore need alternative methods of detection.

Thus, other techniques have included Raman spectroscopy for the chemical imaging of productive clones,^793,794^ while IR spectroscopy has been used to assess secondary structure (*i.e.* folding).^795^ Various constructs have been used to make non-fluorescent substrates or product produce a fluorescence signal.^796^ These include substrate-induced gene expression screening^797–799^ and product-induced gene expression,^800^ fluorescent RNAs,^801^ reporter bacteria,^773,802^ the detection of metabolites by fluorogenic RNA aptamers,^803–811^ colourimetric aptamers and Au particles,^812^ or appropriate transcription factors.^787^ Riboswitches that respond to product formation,^742,743^ chemical tags,^813,814^ and chemical proteomics^815^ have also been used as reporters for the production of small molecules.

Solid-phase screening with digital imaging is another alternative used for the engineering of biocatalysts. These methods generally use microbial colonies expressing the protein of interest to screen for activity directly *in situ*.^816–818^ Advantages to this include the ability to use enzyme-coupled assays (like HRP)^819,820^ or substrates of poor solubility or viscosity.^821^

### Microfluidics, microdroplets and microcompartments

Sometimes the ‘host’ and the screen are virtually synonymous, as this kind of miniaturisation can also offer considerable speeds.^822–825^ Thus, there are trends towards the analysis of directed evolution experiments in microcompartments,^766,826–831^ using suitable microfluidics^777,832–838^ or picodroplets.^831,839–843^ Agresti *et al.* ^844^ have shown that microfluidics using picolitre-volume droplets can screen a library of 10^8^ HRP mutants in 10 hours. Although further refinement of microfluidics-based screening is required before its use becomes commonplace, it is clear that it has the capability to process the larger and more diverse libraries that one wishes to investigate.

## Assessment of diversity and its maintenance

By now we have acquired a population of clones that are ‘better’ in some sense(s) than those of their parents. If we measure only fitnesses, however, as we have implicitly done thus far, we have only half the story, and we now return to the question of using knowledge of where we are or have been in a search space to optimize how we navigate it. There is of course a considerable literature on the role of ‘genetic’ and related searches in all kinds of single and multi-objective optimisation (see *e.g.* ref. 106, 107, 110, 113, 116, 210–213 and 845–858), all of which recognises that there is a trade-off between ‘exploration’ (looking for productive parts of the landscape) and ‘exploitation’ (performing more local searches in those parts). Methods or algorithms such as ‘efficient global optimisation’^859^ calculate these explicitly. Of course ‘where’ we are in the search space is simply encoded by the protein's sequence.

There is thus an increasing recognition that for the assessment^860–863^ and maintenance^864^ of diversity under selection one needs to study sequence-activity relationships. When DNA sequencing was much more expensive, methods were focused on assessing functionally important residues (*e.g.* ref. 865–868). As sequences became more readily available, methods such as PROSAR^219,232,233,869^ were used to fix favourable amino acids, a strategy that proved rather effective (albeit that it does not consider epistasis). Now (although sequence-free methods are also possible^340,870–872^), as large-scale DNA (including ‘next-generation’) sequencing becomes commonplace in DE,^873–876^ we may hope to see large and rich datasets becoming openly available to those who care to analyse them.

### Sequence-activity relationships and machine learning

**Fig. 15:**
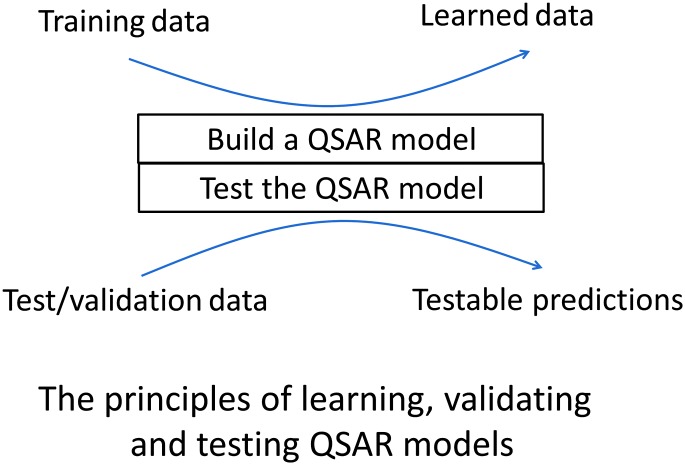
The principles of building and testing a machine learning model, illustrated here with a QSAR model. We start with paired inputs and outputs (here sequences and activities) and learn a nonlinear mapping between the two. Methods for doing this that we have found effective include genetic programming^1345^ and random forests.^23^ In a second phase, the learned model is used to make predictions on an unseen validation and/or test set^1346^ to establish that the model has generalized well.

A historically important development in what is nowadays usually known as machine learning (ML)^877–879^ was the recognition that it is possible to learn relationships (in the form of mathematical models) between paired inputs and outputs – in the classical case between mass spectra and the structures of the molecules that had generated them^880–884^ – and more importantly that one could apply such models successfully in a predictive manner to molecules and spectra not used in the generation of the model. Such models are thus said to ‘learn’, or to ‘generalise’ to unseen samples (Fig. 15).

In a similar vein, the first implementation of the idea that one could learn a mathematical model that captured the (normally rather nonlinear) relationships between a macromolecule's sequence and its activity in an assay of some kind, and thereby use that model to predict (*in silico*) the activities of untested sequences, seems to be that of Jonsson *et al*.^885^ These authors^885^ used partial least squares regression (a statistical model rather than ML – for the key differences see ref. 886) to establish a ‘quantitative sequence-activity model’ (QSAM) between (a numerical description of) 68-base-pair fragments of 25 *E. coli* promoters and their corresponding promoter strengths. The QSAM was then used to predict two 68 bp fragments that it was hoped would be more potent promoters than any in the training set. While extrapolation, to ‘fitnesses’ beyond what had been seen thus far, was probably a little optimistic, this work showed that such kinds of mappings were indeed possible (*e.g.* ref. 887–891). We have used such methods for a variety of protein-related problems, including predicting the nature and visibility of protein mass spectra.^892–894^

As a separate example, we used another ML method known as ‘random forests’^895^ to learn the relationship between features of some 40 000 macromolecular (DNA aptamer) sequences and their activities,^23^ and could use this to predict (from a search space some 14 orders of magnitude greater) the activities of previously untested sequences. While considerable work is going on in structural biology, we are always going to have very many more (indeed increasingly more) sequences than we have structures; thus we consider that approaches such as this are going to be very important in speeding up DE in biocatalysis and improving the functional annotation of proteins. In particular, those performing directed evolution can have simultaneous access to all sequences and activities for a given protein.^896,897^ In contrast, an individual protein undergoing natural evolution cannot in any sense have a detailed ‘memory’ of its evolutionary past or pathway and in any event cannot (so far as is known, but *cf.* ref. 122 and 898) itself determine where to make mutations (only what to select on the basis of a poorly specified fitness). Machine learning methods seem extremely well suited for searching landscapes of this type.^23,56,107,677,899^ Overall, this is a very important difference between natural evolution and (Experimenter-) Directed Evolution.

## The objective function(s): metrics for the effectiveness of biocatalysts

This is not a review of enzyme kinetics and kinetic mechanisms,^549,900–902^ and for our purposes we shall mainly assume that we are dealing with enzymes that catalyse thermodynamically favourable reactions, operating *via* a Michaelis–Menten type of reaction whose kinetic properties can largely be characterized *via* binding or Michaelis constants plus a (slower) catalytic rate constant *k*_cat_ that is equivalent to the enzyme's turnover number (with units of reciprocal time). Much literature (*e.g.* ref. 549, 902 and 903) summarises the view that an appropriate measure of the effectiveness of an enzyme is a high value of *k*_cat_/*K*_m_, effected *via* the transduction of the initial energy of substrate/cofactor binding.^903–905^ Certainly the lowering of *K*_m_ alone is a very poor target for most purposes in directed evolution where initial substrate concentrations are large. Better (as an objective function) than enantiomeric excess for chiral reactions producing a preferred *R* form (preferred over the *S* form) is a *P* factor or *E* factor (*k*_cat,*R*_/*K*_m,*R*_)/(*k*_cat,*S*_/*K*_m,*S*_)^906^ of a product. For industrial purposes, we are normally much more interested in the overall conversion in a reactor, rather than any specific enzyme kinetic parameter. Hence, the space-time yield (STY) or volume–time output (VTO) over a specified period, whose units are expressed in amount × (volume × time)^–1^ (*e.g.* ref. 907–911) has also been preferred as an objective function. This is clearly more logical from the engineering point of view, but for understanding how best to drive directed evolution at the molecular level, it is arguably best to concentrate on *k*_cat_, *i.e.* the turnover number, which is what we do here.

### The distribution of *k*_cat_ values among natural proteins

Not least because of the classic and beautiful work on triose phosphate isomerase, an enzyme that is operating almost at the diffusion-controlled limit,^383,912^ there is a quite pervasive view that natural evolution has taken enzymes ‘as far as they can go’ to make ‘proficient’ enzymes (*e.g.* ref. 913–915). Were this to be the case, there would be little point in developing directed evolution save for artificial substrates. However, it is not; most enzymes operate *in vivo* (and *in vitro*) at rates much lower than diffusion-controlled limits^916,917^ (online databases of enzyme kinetic parameters include BRENDA^918^ and SABIO-RK^919^). One assumes that this is largely because evolution simply had no need (*i.e.* faster enzymes did not confer sufficient evolutionary advantage)^920^ to select for them to increase their rates beyond that necessary to lose substantial flux control (a systems property^921–925^). It is this in particular that makes it both desirable and possible to improve *k*_cat_ or *k*_cat_/*K*_m_ values over what Nature thus far has commonly achieved.

In biotransformations studies, most papers appear to report processes in terms of g product × (g enzyme × day)^–1^; while process parameters are important,^907^ this serves (and is probably designed) to hide the very poor molecular kinetic parameters that actually pertain. *K*_m_ is largely irrelevant because the concentrations in use are huge; thus our focus is on *k*_cat_. While DE has been shown to be capable of improving enzyme turnover numbers significantly, calculations show that even the ‘poster child’ examples (prositagliptin ketone transaminase,^926^ ∼0.03 s^–1^; halohydrin dehydrogenase,^219^ ∼2 s^–1^; isopropylmalate dehydrogenase,^927^ ∼5 s^–1^; lovD,^368^ ∼2 s^–1^) have turnover numbers that are very poor compared to those typical of primary metabolism, let alone the diffusion-controlled rates (depending on *K*_m_) of nearer 10^6^–10^7^ s^–1^.^916,917^

### What enzyme properties determine *k*_cat_ values?

Almost since the beginning of molecular enzymology, scientists have come to wonder what particular features of enzymes are ‘responsible’ for enzyme catalytic power (*i.e.* can be used to explain it, from a mechanistic point of view).^928–930^ It is implausible that there will be a unitary answer, as different sources will contribute differently in different cases. Scientifically, one may assume from the many successes of protein engineering that comparing various related sequences (and structures) by homology will be productive for our understanding of enzymology. Directed evolution studies increase the opportunities massively.

In general terms (*e.g.* ref. 930–933), preferred contributions to mechanisms have their different proponents, with such contributions being ascribed variously to a ‘Circe effect’,^904^ strain or distortion,^934^ electrostatic pre-organisation,^933,935–939^ hydrogen tunneling,^940–947^ reorganization energy,^948^ and in particular various kinds of fluctuations and enzyme dynamics.^254,379,930,942,944–946,949–978^ Less well-known flavours of dynamics include the idea that solitons may be involved.^979,980^ Overall, we consider that the ‘dynamics’ view of enzyme action is especially attractive for those seeking to increase the turnover number of an enzyme.

**Fig. 16:**
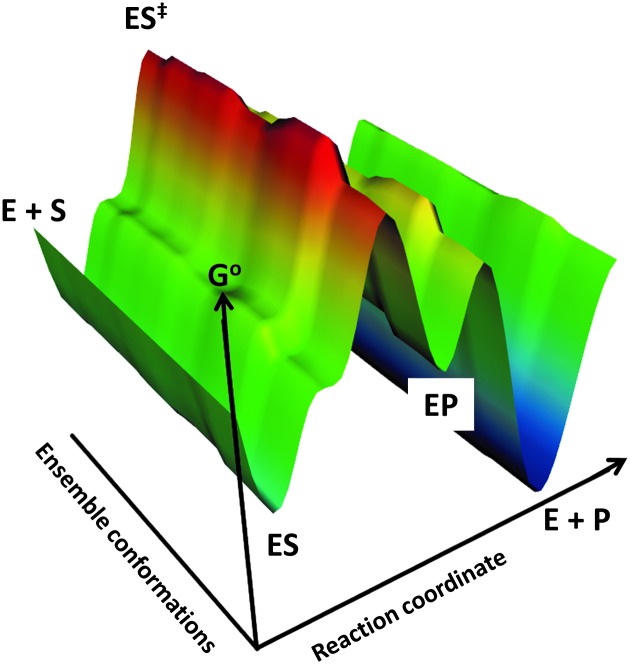
A standard representation of an energy diagram for enzyme catalysis. Substrate binding is thermodynamically favourable, but to effect the catalytic reaction thermal energy is used to take the reaction to the right, often shown as a barrier represented by one or more ‘transition states’. Changes in the *K*_m_ and *K*_d_ (affecting substrate affinity) can be influenced most directly by mutagenesis of the residues at the active site whilst changes in the *k*_cat_ occur primarily from mutagenesis of residues away from the active site (which can affect the fluctuations in enzyme structure required either for crossing the transition state ES^‡^ or by tunnelling under the barrier). At all points there are multiple roughly iso-energetic conformational (sub)states. Figure based on elements of those in ref. 930 and 966.

This is because what is not in doubt is that following substrate binding at the active site (that is dominantly responsible for substrate affinity and the degree of specificity), the binding energy has been ‘used up’ (Fig. 16) and is not thereby available to drive the catalytic step in a thermodynamic sense. This means that the protein must explore its energy landscape *via* conformational fluctuations that are essentially isoenergetic,^951,966,981^ before finding a configuration that places the active site residues into positions appropriate to effect the chemical catalysis itself, that happens as a ‘protein quake’^981,982^ in picoseconds or less.^983^ The source of these motions, whether normal mode or otherwise,^984,985^ can only be the protein and solvent fluctuations in the heat bath, and this means that their origins can lie in any parts of the protein, not just the few amino acids at the active site. Two exceedingly important corollaries of this follow. The first is that one may hope to predict or reflect this through the methods of molecular dynamics even when the active site is essentially unchanged, and this has recently been shown^368^). The second corollary is that one should expect it to be found that successful directed evolution programs that increase *k*_cat_ lead to many mutations that are very distant from the active site. This can also serve, at least in part, to account for why surface post-translational modifications such as glycosylation can have significant effects on turnover (*e.g.* ref. 986).

## The importance of non-active-site mutations in increasing *k*_cat_ values

**Fig. 17:**
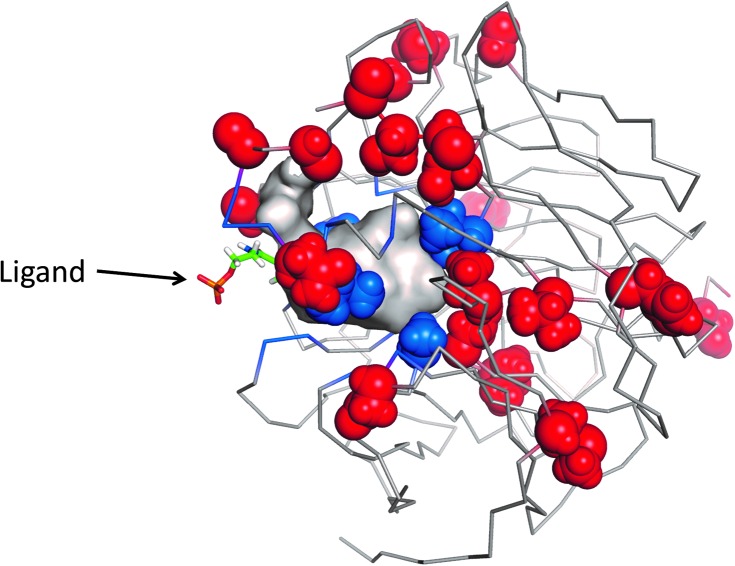
The residues that influence *k*_cat_ tend to be distributed throughout an enzyme. The amino acid side chains of each of the 24 mutations obtained^993^ by the directed evolution of a Dielsalderase (PDB: ; 4O5T) are highlighted. The active site pocket is shown in grey, while all mutated residues within 5 Å of the ligand (blue) are differentiated from those more than 5 Å away (red). This illustrates that the majority of mutations influencing *k*_cat_ are not in close proximity to the active (substrate-binding) site. The figure was prepared using PyMol.

In general terms, it is known that there is a considerable amount of long-range allostery in proteins,^987^ such that distant mutations couple to the active site.^988^ Indeed, most mutations (and amino acid residues) are necessarily distant from the active site, and there is a lack of correlation between a mutation's influence on *k*_cat_ and its proximity to the active site^989^ (by contrast, specificity is determined much more by the closeness to the active site^990^). We still do not have that much data, since we require 3D structural information on many related variants; however, a number of excellent examples (Table 2) are indeed consistent with this recognition that effective DE strategies that raise *k*_cat_ require that we spread our attention throughout the protein, and do not simply concentrate on the active site (Fig. 17). Indeed mutants with major improvements in *k*_cat_ may display only minor changes in active site structure.^368,938,974,991^

**Table 2:** Some examples of improvements in biocatalytic activities that have been achieved using directed evolution, focusing on examples where most relevant mutations are in amino acids that are distal to the active site

Target	Fold improvement over starting point	Ref.	Other notes
Cytochrome P450	9000	56 and 992	20 from generation 5, more than 15 away from active site
Diels–Alderase	*k*_cat_ 108-fold; catalytic power 9000-fold	993	21 aa, 16 outside active site
Glycerol dehydratase	336	994	2 aa, both very distant from active site
Glyphosate acyltransferase	200 in *k*_cat_	995	21 mutations, only 4 at active site
Halohydrin dehalogenase	4000 in volumetric productivity	219	35 mutations, only 8 at active site
3-Isopropylmalate dehydrogenase	65	927	8 mutations, 6 distant from active site
LovD	>1000	368	29 mutations, 18 on enzyme surface
Phosphotriesterase	25	996	7, only 1 at active site
Prositagliptin ketone transaminase	∞ (no starting activity)	926	27 mutations, 17 binding substrate. 200 g L^–1^, >99.5 ee
Triose phosphate isomerase	>10 000	386	36 mutations, only 1 at active site (NB effects on dimerisation, also implying distant effects)
Valine aminotransferase	21 000 000	997	17 mutations, only 1 at active site

## What if we lack the structure? Folding up proteins ‘from scratch’

A very particular kind of dynamics is that which leads a protein to fold into its tertiary structure in the first place, and the purpose of this brief section is to draw attention to some recent advances that might allow us to do this computationally. Advances in specialist computer hardware (albeit not yet widely available) can now make a prediction of how a given (smallish) protein sequence will fold up ‘*ab initio*’.^998–1002^ However, there are many more protein sequences than there are structures, and this gap is destined to become considerably wider^1003^ as sequencing methods continue to increase in speed.^102^ The need for methods that can fold up proteins accurately ‘*de novo*’ (from their sequences) is thus acute.^1004^ However, despite a number of advances (*e.g.* ref. 1000, 1005 and 1006) this is not yet routine. The problem is, of course, that the search space of possible structures is enormous,^1^ and largely unconstrained. As well as using more powerful hardware, the real key is finding suitable constraints. An important recognition is that the covariance of residues in a series of homologous functional proteins provides a massive constraint on the inter-residue contacts and thus what structures they might adopt, and substantial advances have recently been made by a number of groups^197,199,1007–1011^ in this regard. Directed evolution supplies an obvious means of creating and assessing suitable sequences.

## Metals

As mentioned above, many proteins use metals and cofactors to aid the chemistry that they can catalyse, and while we shall not discuss cofactors, a short section on metalloenzymes is warranted, not least since nearly half of natural enzymes contain metals,^1012^ albeit that free metals can be quite toxic.^1013–1015^

To this end, if one wishes to keep open the possibility of incorporating metals into proteins undergoing DE (sometimes referred to as hybrid enzymes^1016–1019^), it is necessary to understand the common mechanisms, residues and structures involved.^460,461,1020–1042^

Some specific and unusual examples include high-valent metal catalysis,^1043^ multi-metal designs as in a di-iron hydrylation reaction,^1044^ a protein whose fluorescence is metal-dependent^1045^ and various chelators, quantum dots and so on^1046–1050^ and metallo-enzymes based on (strept)avidin–biotin technology.^1051–1053^

A particular attraction of DE is that it becomes possible to incorporate metal ions that are rarely (or never) used in living organisms, to provide novel functions. Examples include iridium,^1054^ rhodium^1055^ and uranium (uranyl).^1056,1057^

## Enzyme stability, including thermostability

In general, the rates of chemical reactions increase with temperature, and if we evolve *k*_cat_ to high levels we may create processes in which temperature may rise naturally anyway (and some processes may simply require it^1058^). In a similar vein, protein stability tends to decrease with increasing temperature, and there is commonly^1059–1061^ (though not always^1062^) a trade-off between *k*_cat_ and thermostability, including at the cellular level.^1063^ This relationship depends effectively on the evolutionary pathway followed.^1062^ As discussed above, thermostability may also sometimes (but not always^171–173^) correlate with evolvability,^175^ and is the result of multiple mutations each contributing a small amount.^1064–1069^

Of course the ‘first law’ of directed evolution is that you get what you select for (even if you did not mean to). Thus if thermostability is important one must incorporate it into one's selection regime, typically by screening for it.^1070,1071^ Of course if one uses a thermophile such as *T. thermophilus* then *in vivo* selection is possible, too.^1072^

As rehearsed above, protein flexibility (a somewhat ill-defined concept^87,1073^) is related to *k*_cat_, and most residues involved in improving *k*_cat_ are away from the active site, at the protein surface (where they are bombarded by solvent thermal fluctuations). The connection between flexibility and thermostability is not well understood, and it does not always follow that less flexibility provides greater stability.^1074,1075^ However, one might suppose that some residues that contribute flexibility are most important for (*i.e.* contribute significantly to) thermostability too. This is indeed the case.^989,1076,1077^ Indeed, the same blend of design and focused (thus semi-empirical) DE that has proved valuable for improving *k*_cat_ values seems to be the best strategy for enhancing thermostability too.^1078–1080^

Some aspects of thermostability^1081–1087^ can be related to individual amino acids (*e.g.* an ionic or H-bond formed by an arginine is of greater strength than that formed by a corresponding lysine, or thermophilic enzymes have more charged and hydrophobic but fewer polar residues^1088,1089^). However, some aspects are best based on analyses of the 3D structure,^456,1090,1091^ *e.g.* intra-helix ion pairs^1092,1093^ and packing density.^1068,1094^ Thus, Greaves and Warwicker^1095^ conclude that “charge number relates to solubility, whereas protein stability is determined by charge location”. The choice of which residues to focus on can be assisted (if a structure is available) by looking at the local flexibility *via* methods such as mutability^1096,1097^ or *via B*-factors,^1062,1098,1099^ or *via* certain kinds of mass spectrometry.^1100–1108^ Constraint Network Analysis^1109^ provides a useful strategy for choosing which residues might be most important for thermostability. Unnatural amino acids may be beneficial too; thus fluoro-aminoacids can increase stability.^1110–1112^

To disentangle the various contributions to *k*_cat_ and thermostability, what we need are detailed studies of sequences and structures as they relate to both of these, and published ones remain largely lacking. However, the goal of finding sequence changes that improve both *k*_cat_ and thermostability is exceptionally desirable. It should also be attainable, on the grounds that protein structural constraints that increase the rate of desirable conformational fluctuations while minimizing those that do not help the enzyme to its catalytically active confirmations must exist and will tend to have this precise effect.

Finally, thermal stresses are not the only stresses that may pertain during a biocatalytic process, albeit sometimes the same mutations can be beneficial in both (*e.g.* in permitting resistance to oxidation^1113,1114^ or catalysis in organic solvents^1115^).

## Solvency

While our focus is on evolving proteins, those that are catalyzing reactions are always immersed in a solvent, and we cannot ignore this completely. Although ‘bulk’ measurements of solvent properties are typically unsuitable for molecular analyses of transport across membranes,^269,335,356,362,1116–1119^ it is the case that some of the binding energy used in enzyme catalysis is effectively used in transferring a substrate from a usually hydrophilic aqueous phase to a usually more ‘hydrophobic’ protein phase. In general, the increased mass/hydrophobicity is also accompanied by a changed value for *K*_m_.^916^ This can lead to some interesting effects of organic solvents, and solvent mixtures,^1120^ on the specificity,^1121–1126^ equilibria^1127^ and catalytic rate constants^1128,1129^ of enzymes, for reasons that are still not entirely understood. However, because the intention of many DE programs is the production of enzymes for use in industrial processes, the ability to function in organic solvents is often another important objective function, and can be solved *via* the above strategies.^577^ One recent trend of note is the exploitation of ionic liquids^1130,1131^ and ‘deep eutectic solvents’^1132–1135^ in biocatalysis.

## Reaction classes

Apart from circumstances involving extremely reactive substrates and products, there is no known reason of principle why one might not be able to evolve a biocatalyst for any more-or-less simple (*i.e.* one-step, mono- or bi-molecular) chemical reaction. Thus, one's imagination is limited only by the reactions chosen (nowadays, for a more complex pathway, *via* retrosynthetic and related strategies (ref. 1136–1148)). Given that these are practically limitless (even if one might wish to start with ‘core’ molecules^1149,1150^), we choose to be illustrative, and thereby provide a table of some of the kinds of reaction, reaction class or products for which the methods of DE have been used, with a slight focus on non-mainstream reactions. (Curiously, a convenient online database for these is presently lacking.) Our main point is that there seems no obvious limitation on reactions, beyond the case of very highly reactive substrates, intermediates or products, for which an enzymatic reaction cannot be evolved. Since the search space of possible enzymes can never be tested exhaustively, it is a safe prediction that we should expect this to hold for many more, and more complex, chemistries than have been tried to date, provided that the thermodynamics are favourable.

While the focus of this review is about how best to navigate the very large search spaces that pertain in directed enzyme evolution, we recognize that a number of processes including enzymes evolved by DE are now operated industrially.^327,328^ Examples include sitagliptin,^926^ generic chiral amine APIs,^1151^ bio-isoprene,^1152^ and atorvastatin.^1153^

## Concluding remarks and future prospects

In our review above, we have developed the idea that the most appropriate strategy for improving biocatalysts involves a judicious interplay between design and empiricism, the former more focused at the active site that determines binding and specificity, while the latter might usefully be focussed more on other surface and non-active-site residues to improve *k*_cat_ and (in part) (thermo)stability. As our knowledge improves, design may begin to play a larger role in optimising *k*_cat_, but we consider that this will still require a considerable improvement in our understanding of the relationships between enzyme sequence, structure and dynamics. Thus, protein improvement is likely to involve the creation of increasingly ‘smart’ variant libraries over larger parts of the protein.

Another such interplay relates to the combination of experimental (‘wet’) and computational (‘dry’) approaches. We detect a significant trend towards more of the latter,^519^ for instance in the use^368,1235,1296,1297^ of molecular dynamics to calculate properties that suggest which residues might be creating internal friction^1298,1299^ and hence lowering *k*_cat_. These examples help to illustrate that predictions and simulations *in silico* are likely to play an increasingly important role in predicting strategies for mutagenesis *in vitro*.

The increasing availability of genomic and metagenomic data, coupled to improvements in the design and prediction of protein structures (and maybe activities) will certainly contribute to improving the initialisation steps of DE. The availability of large sets of protein homologues and analogues will lead to a greater understanding of the relationships (Fig. 1) between protein sequence, structure, dynamics and catalytic activities, all of which can contribute to the design of DE experiments. Together with the development of improved synthetic biology methodology for DNA synthesis and variation, the tools for designing and initialising DE experiments are increasing greatly.

Specifically, the availability of large numbers of sequence-activity pairs may be used to learn to predict where mutations might best be tested. This decreases the empiricism of entirely random mutations in favour of synthetic biology strategies in which one has (at least statistically) more or less complete control over which sequences to make and test. Thus we see a considerable role for modern versions of sequence-activity mapping based on appropriate machine learning methods as a means of predicting where searches might optimally be conducted; this can be done *in silico* before creating the sequences themselves.^23^ No doubt many useful datasets of this type exist in the databases of commercial organisations, but they need to become public as the likelihood is that crowdsourcing analyses would add value for their originators^1300^ as well as for the common good.^1301^

In terms of optimisation algorithms, we have already pointed out that very few of the modern algorithms of evolutionary optimisation have been applied to the DE problem,^107^ and the advent of synthetic biology now makes their development and comparison (given that no one size will fit all^1302–1306^) a worthwhile and timely endeavour. Complex DE algorithms that have no real counterpart in natural evolution can also now be carried out using the methods of synthetic biology.

Searching our empirical knowledge of reactions is becoming increasingly straightforward as it becomes digitised. As implied above, we expect to see an increasing cross-fertilisation between the fields of bioinformatics and cheminformatics^1307,1308^ and text mining;^1309–1311^ a very interesting development in this direction is that of Cadeddu *et al*.^1136^

Conspicuous by their absence in Table 3 are the members of one important set of reactions that are widely ignored (because they do not always involve actual chemical transformations). These are the transmembrane transporters, and they make up fully one third of the reactions in the reconstructed yeast^1312^ and human^25,1313^ metabolic networks. Despite a widespread and longstanding assumption (*e.g.* ref. 1314) that xenobiotics simply tend to ‘float’ across biological membranes according to their lipophilicity, it is here worth highlighting the considerable literature (that we have reviewed elsewhere, *e.g.* ref. 269, 335, 356, 357, 362, 1116–1119 and 1315), including a couple of experimental examples (ref. 749 and 750), that implies that the diffusion of xenobiotics through phospholipid bilayers in intact cells is normally negligible. It is now clear that transporters enhance (and are probably required for) the transmembrane transport even of hydrophobic molecules such as alkanes,^1316–1321^ terpenoids,^1319,1322,1323^ long-chain,^1324–1328^ and short-chain^1329–1332^ fatty acids, and even CO_2_.^1333,1334^ This may imply a significantly enhanced role for transporter engineering in whole cell biocatalysis.

**Table 3:** Some reactions, reaction classes or product types for which DE has proved successful. We largely exclude the very well-established programmes such as ketone and other stereoselective reductases, which along with various other reactions aimed at pharmaceutical intermediates have recently been reviewed in *e.g.* ref. 326, 328 and 1154–1162. Chiralities are implicit

Reaction (class) or substrate/product	Illustrative ref.
Aldolases *e.g.* R_1_CHO + R_2_C(=O)R_3_ ⇌ R_1_C(=O)CH_2_C(O)R_3_	462, 1163 and 1164

Alkenyl and arylmalonate decarboxylases *e.g.* HOOCC(R_1_R_2_)COOH → HC(R_1_R_2_)COOH	1165
Amines	1166–1169

Amine dehydrogenase RC(=O)Me + NH_3_ + NADH + H^+^ → RCHNH_2_Me + H_2_O + NAD^+^	1167

Antifreeze proteins	1170

Azidation RH → RN_3_	1171 and 1172

Baeyer–Villiger monooxygenases 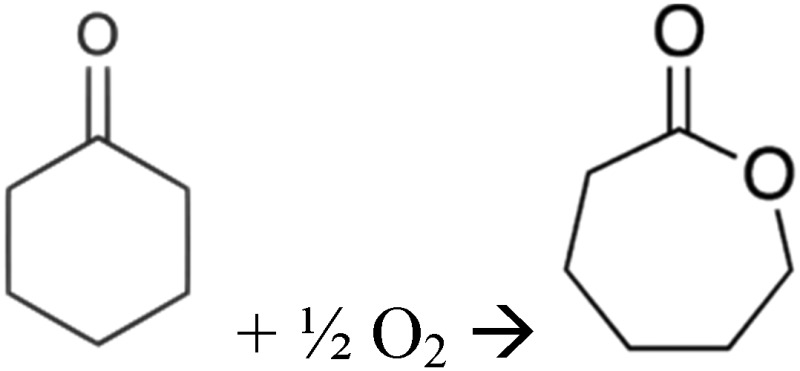	1173 and 1174

Beta-keto adipate HOOCCH_2_CH_2_C(=O)CH_2_COOH	1175

Carotenoid biosynthesis	1176

Chlorinase Ar–H → Ar–Cl	1177–1180

Chloroperoxidase RH + Cl^–^ + H_2_O_2_ → RCl + H_2_O + OH^–^	1181–1187

CO groups	1157

Cytochromes P450 *e.g.* R–H → R–OH	56, 366, 632, 633, 674, 692, 992 and 1188–1208

Diels–Alderases *e.g.* CH_2_ =CHCH=CH_2_ + CH_2_ =CH_2_ → cyclohexene	378, 380, 993, 1209 and 1210

DNA polymerase	1211 and 1212

Endopeptidases	769

Esterase enantioselectivity	1213

Epoxide hydrolase 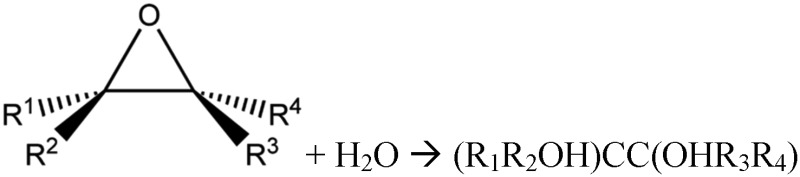	947

Flavanones 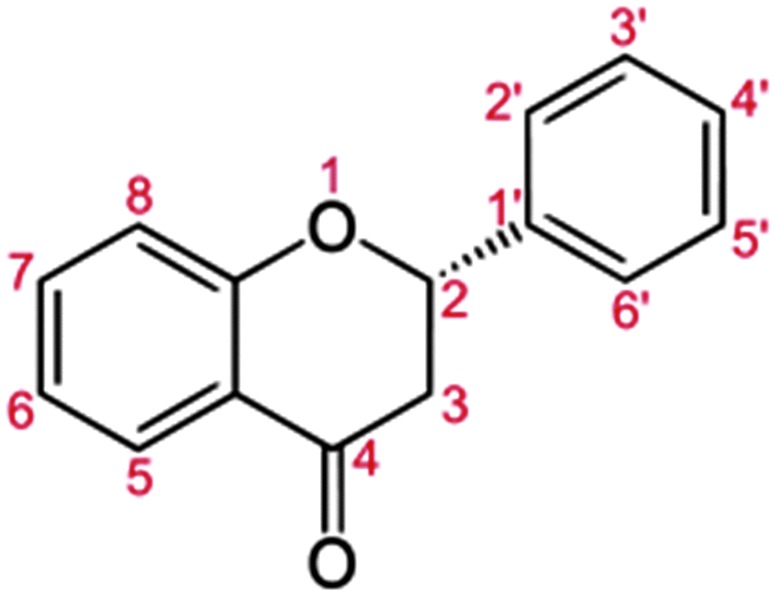	1214–1221

Fluorinase	1178 and 1222 (and see ref. 1223)

Fatty acids	1224–1226

Glyphosate acyltransferase HOOCCH_2_NHCH_2_PO_3_^2–^ + AcCoA → HOOCCH_2_N(CH_3_C=O)CH_2_PO_3_^2–^ + CoA	995 and 1227–1229

Glycine (glyphosate) oxidase HOOCCH_2_NHCH_2_PO_3_^2–^ + O_2_ → OHC–COOH + H_2_NCH_2_ PO_3_^2–^ + H_2_O_2_	1229–1231

Haloalkane dehalogenase R_1_C(HBr)R_2_ + H_2_O → R_1_C(HOH)R_2_ + H^+^ + Br^–^	1232–1235

Halogenase Ar–H → Ar–Hal	1236–1239

Hydroxytyrosol 2–NO_2_–Ph–CH_2_CH_2_OH + O_2_ → 2–OH, 3–OH–Ph–CH_2_CH_2_OH + NO_2_	1240

Kemp eliminase	377 and 1241–1247

Ketone reductions R_1_–C(=O)–R_2_ → R_1_–CH(OH)–R_2_	1248 and 1249

Laccase	1250–1252

Michael addition R–CH_2_CHO + Ph–CH=CHNO_2_ → OHC–CH(R)–CH(Ph)CH_2_NO_2_	1253

Monoamine oxidase R_1_R_2_CHCH_2_NR_3_R_4_ + 1/2O_2_ →R_1_R_2_C=NR_3_ (R_4_ = H) or R_1_R_2_CH=N^+^R_3_R_4_ (R_4_ = alkyl) + H_2_O	728, 1151 and 1254–1256

Nitrogenase N_2_ + 3H_2_ → 2NH_3_	1257

Nucleases	1258

Old yellow enzyme (activated alkene reductions)	664, 1259 and 1260

Paraoxonase R_1_(R_2_O)(R_3_O)–P=O → R_1_(R_2_O)(HO)P=O + R_3_OH	1261–1263

Peroxidase	1264 and 1265

Phospho(mono/di/tri)esterases	255, 831 and 1266–1271

Polyketides	1272–1275

Polylactate	1276

Redox enzymes	1277–1279

Reductive cyclisation	1280

Restriction protease	1281

Retro-aldolase *e.g.* R_1_C(=O)CH_2_C(O)R_3_ ⇌ R_1_CHO + R_2_C(=O)R_3_	463 and 1282

Sesquiterpene synthases	1283

Tautomerases Ar–CH=C(OH)COOH ⇌ Ar–CH_2_COCOOH	1284

Terpene synthase/cyclase	1285–1287

Transaldolase erythrose-4-phosphate + fructose-6-phosphate → glyceraldehyde-3-phosphate + sedoheptulose-7-phosphate	1288 and 1289

Transketolase RCHO + HOCH_2_COCOOH → RCH(OH)COCH_2_OH	1290–1293

Zinc finger proteins	1294 and 1295

The recent introduction of the community standard Synthetic Biology Open Language (SBOL) will certainly facilitate the sharing and re-use of synthetic biology designed sequences and modules. Beginning in 2008, the development of SBOL has been driven by an international community of computational synthetic biologists, and has led to the introduction of an initial standard for the sharing of synthetic DNA sequences^1335^ and also for their visualisation. A recent proposal has introduced a more complete extension to the language, covering interactions between synthetic sequences, the design of modules and specification of their overall function.^1336^ Just as with the Systems Biology Markup Language,^1337^ the Systems Biology Graphical Notation,^1338^ and related controlled vocabularies, metadata and ontologies for knowledge exchange in systems biology^1339,1340^ and metabolomics,^1341^ the availability of these kinds of standards will help move the field forward considerably.

Overall, we conclude that existing and emerging knowledge-based methods exploiting the strategies and capabilities of synthetic biology and the power of e-science will be a huge driver for the improvement of biocatalysts by directed evolution. We have only just begun.
